# How People Understand Risk Matrices, and How Matrix Design Can Improve their Use: Findings from Randomized Controlled Studies

**DOI:** 10.1111/risa.13822

**Published:** 2021-09-14

**Authors:** Holly Sutherland, Gabriel Recchia, Sarah Dryhurst, Alexandra L.J. Freeman

**Affiliations:** ^1^ Winton Centre for Risk and Evidence Communication, Department of Pure Mathematics and Mathematical Statistics University of Cambridge Cambridge UK; ^2^ Salvesen Mindroom Research Centre University of Edinburgh Edinburgh UK

**Keywords:** Consequence, impact, likelihood, probability, risk communication, risk matrix

## Abstract

Risk matrices are a common way to communicate the likelihood and potential impacts of a variety of risks. Until now, there has been little empirical work on their effectiveness in supporting understanding and decision making, and on how different design choices affect these. In this pair of online experiments (total *n* = 2699), we show that risk matrices are not always superior to text for the presentation of risk information, and that a nonlinear/geometric labeling scheme helps matrix comprehension (when the likelihood/impact scales are nonlinear). To a lesser degree, results suggested that changing the shape of the matrix so that cells increase in size nonlinearly facilitates comprehension as compared to text alone, and that comprehension might be enhanced by integrating further details about the likelihood and impact onto the axes of the matrix rather than putting them in a separate key. These changes did not affect participants’ preference for reducing impact over reducing likelihood when making decisions about risk mitigation. We recommend that designers of risk matrices consider these changes to facilitate better understanding of relationships among risks.

## 1 INTRODUCTION

In order to prioritize resources and make decisions when mitigating or preparing for hazards, risk managers and responders need to understand those hazards’ likelihoods and potential impacts. A common way to represent these is via a risk matrix: a table or graph illustrating likelihood on one axis and potential impact on the other. Hazards are then graphically represented by being located in the appropriate section of the matrix space. This allows an “at a glance” assessment of the risk a given hazard poses and of the potential effects of mitigation measures, and a comparison between it and other hazards in the matrix.

In addition to facilitating decision making by risk managers, such matrices are also used to communicate information to policymakers and sometimes the broader public. For example, the U.K. National Risk Register, an accounting of malicious and nonmalicious risks facing the United Kingdom and their estimated likelihood and impact, helps guide government decision making. The public version of this register includes a risk matrix, and information is presented to policymakers and stakeholders in a similar format (The U.K. Cabinet Office, 2017). There are also other contexts where risk matrices are used for decision making by individuals who are not necessarily frequent or sophisticated users of the format, such as when regulatory or compliance guidelines mandate their use for risk assessment or mitigation plans that occur on a relatively infrequent basis. In some such cases, they are being used by individuals who use such matrices rarely, and for whom risk assessment or mitigation may only be a small and occasional part of their job. It is therefore crucial to ensure that risk matrices are clear and comprehensible for nonexpert users.

Risk matrices find widespread use in many critical contexts, such as national risk registers, reports by national and international organizations, international safety standards, and military risk assessments (see Dillon, Liebe, & Bestafka, 2009; Kerckhoffs, 2017; The U.K. Cabinet Office, 2017; United State Department of Defense, 1993; World Economic Forum, 2021 and many of the International Organization for Standardization standards for examples). In some cases, both of the matrix's axes may be numerically specified and the risks well‐quantified (quantitative risk matrices), but equally matrices may be used to communicate much more qualitative assessments of risk, with either one or both axes being categorical (semiqualitative and qualitative risk matrices). In almost all cases, however, the axes represent nonlinear scales. In this project we took as an exemplar the U.K.’s National Risk Register where, as in many organizations, the risks are semi‐qualitative: likelihoods of reasonable worst‐case scenarios are expressed in one of five categories, as are their corresponding impacts. Likelihood categories are pinned to probabilistic ranges, for example, “25 to 125 in 500,” while the impact scale is ordinal. In addition, the likelihood categories represent logarithmically increasing probabilities, with each category representing a likelihood range five times greater than the previous.

There are extensive theoretical critiques of risk matrices (Cox, Babayev, & Huber, 2005; Cox, 2008; Duijm, 2015; Monat & Doremus, 2018), most of which are aimed at mathematical or logical issues with quantitative risk matrices; there appears to be little literature considering the merits and demerits of qualitative and semiqualitative matrices. There is some qualitative experimental evidence that people may struggle to use semiqualitative risk matrices to make rational decisions (Monat & Doremus, 2018), but there is otherwise also an absence of experimental studies on people's perception, use, and comprehension of risk matrices. While in many cases risk matrices are used as a prompt during the risk assessment process to help risk managers think through the potential outcomes of different events and the potential mitigations they might put in place, in other cases risk matrices are used specifically to attempt to communicate a risk manager's assessment to another individual, to allow the latter to consider the hazards and weigh up potential mitigation activities.

### 1.1 Room for Improvement?

The use of logarithmically increasing categories in risk matrices is very common and is sometimes explicitly recommended (Wijnia, 2012). Logarithmic scales facilitate comparison between risks that span several orders of magnitude, and can aid interpretation of data with logarithmic/exponential trends (Field, 1917). However, research suggests that even expert audiences have difficulties with logarithmic scales. For example, in a survey sent to the United States’ largest organization of professional ecologists, far fewer respondents correctly interpreted graphs with log‐log scales than graphs with linear scales (56% versus 93%, respectively, Menge et al., 2018). Significant difficulties in understanding plots with logarithmic axes have also been demonstrated among third and fourth‐year engineering undergraduates (Heckler, Mikula, & Rosenblatt, 2013) and the general public (Romano, Sotis, Dominioni, & Guidi, 2020).

If audiences do not comprehend the nonlinear nature of risk matrix axes, this may considerably compromise their ability to make decisions that involve comparing risks posed by different hazards (or a hazard before and after mitigation), the very decisions that risk matrices are designed to assist with. Whether there are changes to risk matrices that make such misinterpretations less likely has never been formally tested. We identified three characteristics of risk matrices, particularly salient in the U.K.’s National Risk Register, that could plausibly affect the degree to which audiences successfully understand the nonlinear scale and manage to keep it in mind while using the matrix: scale labeling, cell shape, and key placement.

#### 1.1.1 Scale Labeling

Some matrix designs, such as the U.K.’s 2017 National Risk Register (The U.K. Cabinet Office, 2017) and the U.S. Centers for Disease Control influenza risk register (Centers for Disease Control and Prevention, 2020), use linear labeling on the axes (e.g., 1, 2, 3, etc.) even though the likelihoods or impacts represented by these labels increase at a nonlinear rate. In light of their findings on expert misunderstanding of log scales, Menge et al. recommended using untransformed values as scale labels (Menge et al., 2018). However, the probabilities in risk matrices are sometimes so small that completely untransformed values may be difficult to interpret; Heckler et al. (Heckler et al., 2013) reported that negative exponents (e.g., 10^−4^) on log scales posed particular challenges for understanding. One possible alternative is presenting more familiar numbers that increase at a nonlinear rate. For example, in a matrix that represents five different levels of likelihood, with each being five times more likely than the previous, labeling the bins with a “geometric” scale of geometrically increasing values 1, 5, 25, 125, and 625 might communicate the relationships between the different levels more clearly than values of 1, 2, 3, 4, and 5.

#### 1.1.2 Cell Shape

In addition to modified scale labeling, adding log tick marks with variable spacing has been shown to help students understand plots with logarithmic axes (Heckler et al., 2013), but this only makes sense for axes that represent continuous values. It may be possible to achieve a similar effect for categorical matrices by modifying the spacing of the lines that divide rows and columns, such that cells to the top‐right of the matrix are larger than those to the bottom‐left. Increasing the size of cells as one moves to the top right of such a matrix—a “logarithmic” format—could have a positive influence on users by providing a visual cue that works with both magnitude priming effects (where the visual size of something can prime a scale‐independent sense of magnitude, Oppenheimer, LeBoeuf, & Brewer, 2008) and size congruity effects (where incongruencies between physical size and numerical value are thought to produce cognitive interference, e.g. Tzelgov, Meyer, & Henik, 1992). In addition, the visual cue may simply serve as a salient reminder that differences between values become larger as one moves further to the top‐right. For example, in a hypothetical matrix where the columns correspond to likelihoods of 0.001%, 0.01%, 0.1%, 1%, and 10%, the difference between columns 1 and 2 is numerically smaller than the difference between columns 4 and 5, a fact which this approach would also represent graphically.^1^ A review of the graph comprehension literature similarly cites ample evidence that “when a visual feature does not automatically evoke a particular fact or relationship, then that information is more difficult to comprehend and viewers may make an error in interpretation” (Shah & Hoeffner, 2002, p. 50), further suggesting that this approach may yield comprehension benefits.

#### 1.1.3 Key Placement

Risk matrices differ in terms of whether information about the meaning of each column and row—the “key” — is integrated directly into the matrix axes or represented separately, in a legend or surrounding text. As legends require the diagram user to reference information away from the diagram itself, the user must keep either the legend (while looking at the diagram) or the diagram (while looking at the legend) in working memory, increasing the cognitive demand of understanding the information provided or of using the diagram (Shah & Hoeffner, 2002). If participants cannot keep the information in working memory, or if they are using the diagram for an extended period of time, they may need to look repeatedly back and forth between the diagram and the legend (Fausset, Rogers, & Fisk, 2008). As such, design guidelines for diagrams or other kinds of visual information generally recommend that as much information be integrated into the diagram as possible, for ease of use (Carpenter & Shah, 1998). This frees up the user's working memory—and other associated executive functioning/cognitive resources—to be used exclusively on deciphering the diagram itself and comprehending/manipulating the information contained therein. However, directly labeling each row and column can also contribute to visual clutter (Shah & Hoeffner, 2002), which may impose its own demands on working memory.

### 1.2 Do Risk Matrix Features Affect Users’ Prioritizations of Likelihood Versus Impact?

Modifications to risk matrices could theoretically also influence perception and prioritization of the two dimensions represented (impact and likelihood). This view is shared by Woodruff (Woodruff, 2005), who, in a critique of a perceived institutional overemphasis on impact over likelihood, argued for the use of a matrix that overlays information about whether risks should be considered acceptable, tolerable, or unacceptable. However, one aspect of the specific matrix Woodruff proposed might unintentionally make impact *more* salient. Specifically, the longest side of Woodruff's proposed matrix is against the axis that was used to represent impact. The physical distance between high‐impact and low‐impact cells thus tends to be larger than the distance between high‐likelihood and low‐likelihood cells. Differences in impact might therefore appear “larger” than differences in likelihood in this matrix, which could theoretically make users more apt to prioritize impact than they would have been if the longest side had been placed against the axis used to represent likelihood,^2^ for example, due to magnitude priming.

### 1.3 Hypotheses

Owing to the considerations in Section 1.1, we hypothesized there would be a difference with respect to participants’ performance on a set of questions we devised that required them to compare multiple risks and to understand the nonlinear nature of the matrix's axes. Specifically, we expected better performance in conditions that emphasized the nonlinear nature of the values represented on the matrix's axes. These conditions were the “logarithmic” format (section 1.1.2), tested in Experiments 1 and 2, and the “geometric” scale (Section 1.1.1), tested in Experiment 2 only.

We also predicted that there would be a difference between conditions in participants’ prioritization of impact versus likelihood reduction. Specifically, we hypothesized that any tendency to reduce impact over likelihood or vice versa would be minimized in conditions with square cells. This hypothesis was tested in Experiment 1.

Both experiments were preregistered, and measures were informed by a pilot study and on qualitative interviews with professional risk managers who use risk matrices during their work. The preregistration for Experiment 1 can be found at https://osf.io/h2s4g with a postpilot amendment at https://osf.io/qf8mg. The preregistration for Experiment 2 can be found at https://osf.io/c93mf. Ethical oversight was given by the Psychology Research Ethics Committee at the University of Cambridge (PRE.2019.023).

## 2 EXPERIMENT 1

### 2.1 Method

#### 2.1.1 Design and Stimuli

**Fig 1:**
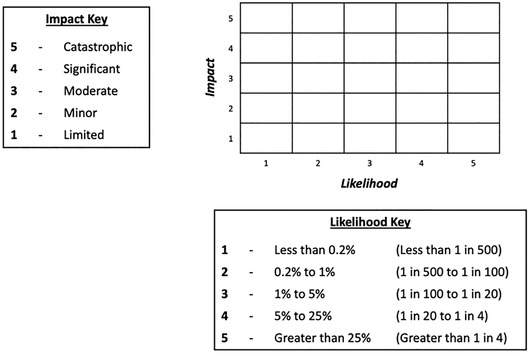
Example of stimulus shown in the standard risk matrix condition

**Fig 2:**
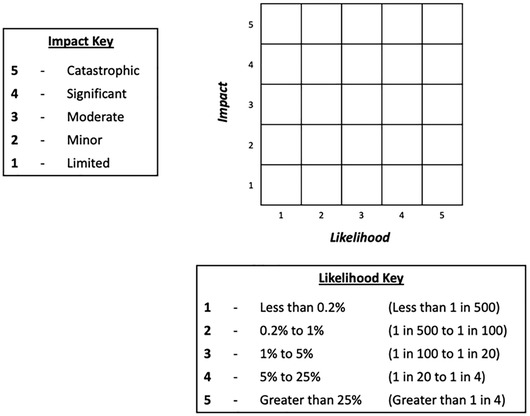
Example of stimulus shown in the square risk matrix condition

**Fig 3:**
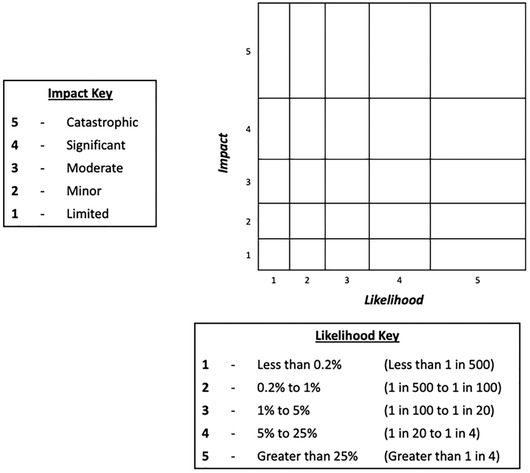
Example of stimulus shown in the “logarithmic” risk matrix condition

**Fig 4:**
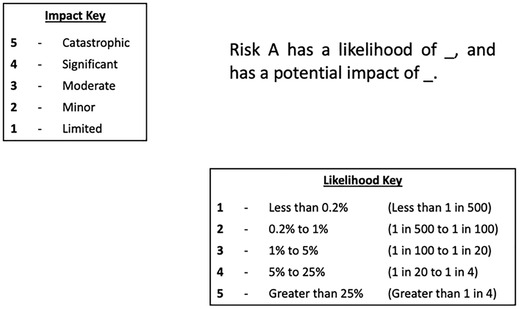
Example of stimulus shown in the text control condition

The aim of this experiment was to assess whether changes to the form of the matrix and its cells affected participants’ basic knowledge (of the likelihoods and impacts of different risks) and use of the information in risk comparisons (e.g., being able to compare effects of risk‐mitigation actions). Participants were randomized to one of four conditions: a standard risk matrix, with rectangular cells (Fig. 1); a risk matrix with square cells (Fig. 2); a “logarithmic” risk matrix designed by the authors, where the width and height of the cells increased as the values on the axes increased (Fig. 3); or the control condition, a short paragraph of text (Fig. 4).

Participants were informed that the risks in question related to hypothetical flood risks, and specific risks labeled with each stimulus were described simply to participants as “risk A” and “risk B”. These abstract terms were intentionally selected so as to avoid potential confounds that any particular choice of concrete flood risks might have elicited. In all cases, risks A and B were assigned to different cells, with each corresponding to a different combination of likelihood and impact. The specific cells in which risks A and B were placed differed for each type of question block; details are provided in Section 2.1.3. There were five possible likelihood categories (less than 0.2% / less than 1 in 500; 0.2−1% / 1–5 in 500; 1−5% / 5–25 in 500; 5−25% / 25–125 in 500; greater than 25% / greater than 125 in 500), corresponding to the likelihood ranges used by the U.K. National Risk Register. In addition, there were five possible impact categories. While the U.K. National Risk Register does not make use of descriptive terms for categorizing impact, we wished to ensure that the increasing level of impact was clear to our lay participants, and therefore employed corresponding category labels in use elsewhere in U.K. government: “limited,” “minor,” “moderate,” “significant,” and “catastrophic” (Fire and Rescue Service, 2020). Stimuli were kept consistent between Experiment 1 and Experiment 2 with the exception of the differences highlighted in the description of Experiment 2. Original stimuli are available in the OSF repository at https://osf.io/es72a/?view_only=7aeef3eceaa743059f64b414eea76159.

#### 2.1.2 Participants

Participants were recruited via the ISO‐accredited market research company Respondi to an online survey presented in Qualtrics. Quotas were used to ensure participants were representative of the U.K. population by sex and age, according to the U.K. census (Office for National Statistics, 2016). Participants were excluded for noncompletion. No outlier participants or data points were excluded. A pilot study (*n* = 142) was used to test the procedure and stimuli (results unreported). The questionnaire was completed in a median time of 23.5 minutes; participants were paid £1.40. Demographic information is reported in Supporting Information Table F1.

To determine the number of participants to recruit, we performed power calculations using G*Power (Faul, Erdfelder, Buchner, & Lang, 2009), aiming for 90% power to detect a small effect size of *f =* 0.1 (Cohen's *d =* 0.2), with an alpha of 0.05. These analyses suggested it would be necessary to recruit *n* = 1,422 participants. Type I error rate was controlled by selecting a small number of hypotheses, rather than by alpha adjustment. Due to quota‐related rounding, 1,426 participants completed the study and were included in the analysis.

#### 2.1.3 Procedure

Participants’ prior experiences and beliefs about flooding, and their experiences with risk matrices, were collected via a series of questions within the survey before they saw any stimuli. They were then given an example matrix (or text control) with instructions on how to read and interpret the condition that they were randomized to (see Appendix A), and were asked to rate how easy they found it to understand, and how useful they thought it would be for making decisions about flood risks, on seven‐point Likert scales. Each participant was then shown basic knowledge, risk comparison, and likelihood versus impact reduction question blocks, as follows:

##### 2.1.3.1 Basic knowledge

Basic knowledge questions were intended to measure participants’ basic comprehension of the information (e.g., their ability to identify correctly the likelihood and impact of a particular risk; their ability to identify which of two risks was more likely or more impactful and by how much). Examples include “What is the likelihood of risk A?,” “Which risk has the greater impact: risk A, or risk B?,” and “Which position on the grid above represents the greatest possible risk?” See Supporting Information Fig. G1 for the full list. The values of risks A and B were randomly sampled from the five likelihood and impact categories, and then checked by the researchers to ensure that a broad range of impact and likelihood combinations occurred within each question block. A ceiling effect was expected on these questions, given their relatively basic nature. The number of correctly answered questions in this block was summed for each participant to create a single ‘basic knowledge score’ out of eight.

##### 2.1.3.2 Risk comparison

A set of more challenging comprehension questions was devised, each of which required participants to engage in a *risk comparison*: that is, to compare multiple risks in a manner that required participants to understand the nonlinear nature of the matrix's axes. Examples include “Which risk has had its likelihood decreased the most?,” “Which risk has had its potential impact decreased the most?,” and “Do you decrease the likelihood of risk A or risk B?,” presented after vignettes about local government efforts to reduce flood risks. See Supporting Information Fig. G2 for the full list and corresponding vignettes. The researchers hand‐selected the position of risks A and B, as there were a limited number of positions on the matrix where A and B could be placed such that the questions would have answers that could be objectively judged as correct or incorrect. There were 18 questions in total and the number of correctly answered questions was summed for each participant to create a single “risk comparison score” out of 18.

##### 2.1.3.3 Likelihood versus impact reduction questions

Participants had three opportunities to indicate whether they would prefer to reduce impact or likelihood when a risk was placed such that participants could either reduce its impact (i.e., move it one cell to the left) or its likelihood (i.e., move it one cell down). The question used in all cases was: “A city in the UK is at risk of flooding, shown above as risk A. The local government has some money it can use to decrease either the likelihood or impact of risk A, by the amount shown. Both options cost the same, and the local government can only afford to choose one. Do you think the local government should choose to decrease the likelihood or the impact of risk A?” Participants were asked the question for risks appearing at <*likelihood category*, *impact category*> coordinates of <2, 4>, <4, 2>, and <4, 4>. Preferences for prioritizing likelihood were scored as −1, preferences for prioritizing impact were scored as 1, and response values were averaged for each participant to provide a “prioritization score.” A score closer to −1 indicated a preference toward prioritizing the reduction of likelihood; a score closer to 1 indicated a preference toward decreasing impact.

The order of question sets^3^ was randomized within each block, and the order of questions within each question set was also randomized. After completing the above tasks, participants again rated the risk presentation format they had been randomized to on how easy it was to understand, how useful they thought it would be for making decisions about flood risks, how confident they would feel using it to make decisions about flood risks, and how confident they would feel using it to explain flood risks to others, on seven‐point Likert scales.

In the final section, participants completed a “matrix preference task” in which they were presented with the four conditions on a single page, and asked to select which risk presentation format they would prefer to use for making decisions about risks. Finally, they completed two questionnaires:

##### 2.1.3.4 Numeracy

Participants completed the adaptive Berlin numeracy test (Cokely, Galesic, Schulz, Ghazal, & Garcia‐Retamero, 2012) and the Schwartz numeracy scale (Schwartz, Woloshin, Black, & Welch, 1997). Combining the Berlin numeracy test with “at least one other test” of numeracy is recommended for general population samples to reduce skew and provide better discrimination between low‐numeracy participants (Cokely et al., 2012), and subsequent work has demonstrated that the Schwartz is a good choice (Cokely, Ghazal, Galesic, Garcia‐Retamero, & Schulz, 2013; Cokely, Ghazal, & Garcia‐Retamero, 2014). We also included a single item from the expanded numeracy scale Lipkus, Samsa, & Rimer (2001): “Which of the following numbers represents the biggest risk? 1 in 100, 1 in 1000, or 1 in 10?”), which has been used alone as a basic measure of objective numeracy (e.g., Nelson, Moser, & Han, 2013). The Berlin numeracy test score was summed with the total number of correct answers on the additional questions to give each participant a “total numeracy score” out of eight.

##### 2.1.3.5 Demographics

In order to assess how representative our survey pool was of the U.K. population, participants were asked their gender, age, race/ethnicity, highest completed education, annual household income for the previous year, and political views from left‐ to right‐wing using a seven‐point scale.

#### 2.1.4 Pilot study

A preregistered pilot study (*n* = 142) was run prior to Experiment 1 to test the measures, and to ensure normality of data for key measures where a ceiling effect was not expected (i.e., the risk comparison score and the prioritization score). A postpilot amendment noting changes made in the light of the results was registered at https://osf.io/qf8mg. Deviations from preregistrations appear in Appendix B.

### 2.2 Results

#### 2.2.1 Hypothesis 1—Risk Comparison Score

Because of significant violations of homogeneity of variance, a partial proportional odds ratio model was fitted to determine the effect of format on risk comparison scores, controlling for numeracy by treating participants’ total numeracy score as a covariate. The text condition was treated as the control (reference group).

Risk comparison scores were higher for participants who had viewed the logarithmic matrix as opposed to the text control (est. = 0.26 (95% CI = 0.01−0.51), *p* = 0.038), confirming Hypothesis 1. There was no significant difference between scores of those who viewed the standard matrix versus the text control (est. = 0.09 (95% CI = −0.14−0.36), *p* = 0.484) or between the square matrix and the text control (est. = 0.01 (95% CI = −0.23−0.25), *p* = 0.935). See Table I for means and standard deviations. Numeracy was a significant covariate.

**Table I:** Means and Standard Deviations of Risk Comparison Scores (out of 18), Basic Knowledge Scores (out of 8), and of Overall Score (out of 26) by Condition

	Format			
	Standard Matrix (*n* = 314)	Square Matrix (*n* = 364)	Logarithmic Matrix (*n* = 362)	Text (*n* = 359)
Risk Comparison Score /18	7.60 (3.19)	7.20 (3.10)	7.75 (3.14)	7.21 (3.82)
Basic Knowledge Score /8	6.80 (1.86)	6.71 (1.84)	6.82 (1.81)	6.48 (2.06)
Overall Score /26	14.40 (4.22)	13.91 (4.27)	14.57 (4.15)	13.69 (5.05)

#### 2.2.2 Hypothesis 2—Likelihood Versus Impact

A one‐sample *t‐*test, with a reference value of 0, revealed a mean prioritization score of 0.09 (95% CI: 0.06 – 0.12), showing that participants indeed demonstrated prioritization toward reducing impact over likelihood, *t*(1426) = 6.25, *p* < 0.0001. An ANCOVA was conducted to determine an effect of format, controlling for numeracy. The ANCOVA showed no main effect of format (*F*_3,1421_ = 0.88, *p* = 0.452). Numeracy was not a significant covariate.

#### 2.2.3 Other Analyses

Apart from our two main, preregistered hypotheses, we also explored whether participants’ comprehension was affected by the amount of previous experience they had with risk matrices. We further considered their subjective preferences between the formats, and explored interactions between format and numeracy. These analyses are reported in Appendix C.

Given the (expected) ceiling effect seen across all conditions on basic knowledge questions, basic knowledge and risk comparison scores for each participant were summed to an overall performance score (out of 26). This was to produce a more normal distribution, and also because both measures were essentially testing how well people understood and could use the condition they had been assigned. As serious violations of homogeneity of variance persisted, a partial proportional odds ratio model was again fitted to determine the effect of format on overall score, controlling for numeracy and using the text condition as the reference group. The logarithmic matrix again performed significantly better than the text control (est. = 0.30, 95% CI = 0.05−0.35, *p* = 0.021); means and standard deviations appear in Table I. There was no significant difference between the standard matrix and the text control (*p* = 0.356), or between the square matrix and the text control (*p* = 0.513).

### 2.3 Discussion

We had hypothesized that the “logarithmic” design of matrix would help emphasize the nonlinear nature of the matrix axes, and hence, compared to a text‐only control, would improve participants’ performance on the risk comparison questions, which required them to understand the magnitude of the differences between risks in different cells. Our results suggested that this was indeed the case. However, this effect was small, and the other forms of risk matrix did not significantly improve participants’ performance over the text‐only control condition. This may be because the information presented was relatively simple, with only one or two risks illustrated per matrix. It is possible that the logarithmic design improved performance because it provided a subtle reminder of the nonlinearity—the presumed mechanism behind the improvements Heckler et al. (2013) found by using log tick marks—but did not actually induce magnitude priming effects. There was no effect of format for participants who already had experience with risk matrices, although there were few enough such participants that our study may have been underpowered to detect effects in this subset.

## 3 EXPERIMENT 2

In Experiment 2, we aimed to replicate the finding from Experiment 1 of better risk comparison scores in participants viewing the logarithmic design than in the text control. Additionally, we aimed to investigate the effects of how the nonlinear scale was represented on the axis labels (with label values that increased linearly: 1, 2, 3, 4, 5 versus geometrically: 1, 5, 25, 125, 625; Section 1.1.1) and of using a legend versus integrating this information directly into the axes, as described in Section 1.1.3. As in Experiment 1, we hypothesized that conditions which emphasized the nonlinear nature of the underlying scale would facilitate risk comparisons. This meant that while we again expected improvements for participants viewing the logarithmic design (vs. the standard design or text only), we also expected improvements for participants viewing geometrically increasing (rather than linearly increasing) scale labels. We further expected that integrating the information about the meaning of each row and column from the keys onto the axes, rather than in a separate legend, would make it more salient and would similarly improve performance.

One drawback of the matrix stimuli used in Experiment 1 was the use of the so‐called “1‐in‐X” format, for example, expressing a risk of 0.2% to 1% as “1 in 500 to 1 in 100.” Although often historically used in natural hazard communication (such as “return periods” for floods and storms), this format has long been known to create confusion, as more people misinterpret which of two risks is higher when they are presented in this format, as opposed to a format where the denominators are kept the same (Grimes & Snively, 1999). This may have had a negative impact on comprehension. The 1‐in‐X representations were therefore revised in Experiment 2 to a “frequency format” with all denominators fixed at 500, the lowest common denominator in this case.

### 3.1 Method

#### 3.1.1 Design and Stimuli

Participants were randomized to one of 12 conditions in a 3×2×2 (format × scale labeling × key) factorial design, with the factors defined as follows:

##### 3.1.1.1 Format

The formats tested were the *standard* risk matrix, “*logarithmic*” risk matrix, and *text* control formats used in Experiment 1.

##### 3.1.1.2 Scale labeling

Two approaches to labeling the axes were tested, *linear* and *geometric*. Participants in the *linear* condition were shown cells labeled from 1 through 5. Participants in the *geometric* condition were shown cells labeled with the numbers 1, 5, 25, 125, and 625, numerically representing the five‐fold scaling of magnitude. Participants in both conditions were informed that each level of the scale represented a magnitude five times greater than the previous level.

##### 3.1.1.3 Key

**Fig 5:**
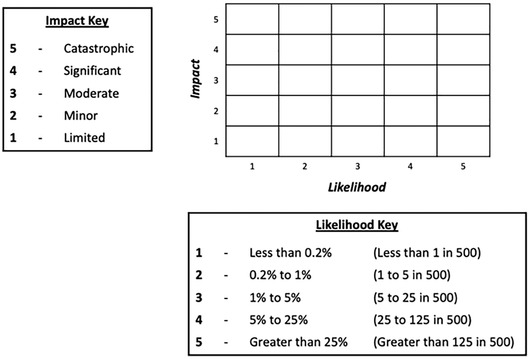
An example of the legend key condition, combined with the standard risk matrix format and linear scale labeling

**Fig 6:**
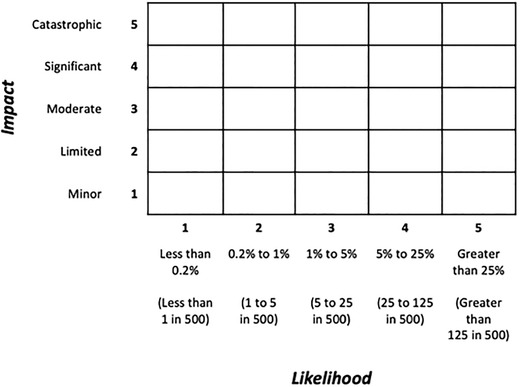
An example of the integrated key condition, combined with the standard risk matrix format and linear scale labeling

We tested two approaches to representing the key. In the *legend* condition (Fig. 5), two legends were placed to the left of and below the matrix/text, providing additional interpretive information about impact and likelihood values respectively. The *integrated* condition (Fig. 6) included additional interpretive information about impact and likelihood values integrated into the matrix axes/body of the text to represent a practical alternative which might give more information to the audience to help them interpret the nonlinear nature of the scales and affect their perception and decision making. In both designs of key, the information given was the same: numerical and frequency ranges for each level of the likelihood axis (less than 0.2% or less than 1 in 500; 0.2−1% 1–5 or in 500; 1−5% or 5–25 in 500; 5−25% or 25–125 in 500; greater than 25% or greater than 125 in 500), and a verbal description for each level of the impact axis (“limited,” “minor,” “moderate,” “significant,” “catastrophic”). To see what stimuli looked like for every combination of format, scale labeling, and key, please see Appendix D.

#### 3.1.2 Participants

Participants were recruited as described in Experiment 1. The same exclusion criterion was used; additionally, participants who took part in Experiment 1 were barred from participating in Experiment 2. Power calculations conducted in the manner previously described suggested it would be necessary to recruit *n* = 1,269 participants. Due to quota‐related rounding, 1,273 participants completed the study and were included in the analysis. Demographic information is reported in Supporting Information Table F1. Experiment 2 was completed in a median time of 22.4 minutes; participants were paid £2.20.

#### 3.1.3 Procedure

The procedure was as in Experiment 1, with two differences. First, a questionnaire coding error meant that two basic knowledge questions (“Which … represents the greatest/smallest risk?”) were not displayed correctly to most participants. Therefore, these two questions were excluded for all participants from all analyses for Experiment 2, leaving six basic knowledge questions overall. Second, in the matrix preference task, participants were asked to choose between the following in three separate questions: a standard matrix, a “logarithmic” matrix, and a short paragraph of text, using the same scale labeling and key conditions they had seen; linear and geometric scale labels using the same format and key conditions they had seen; and a separate legend versus integrated information using the same format and scale labels they had seen.

### 3.2 Results

#### 3.2.1 Risk Comparison Scores

A three‐way ANCOVA was conducted to determine the effect of format, scale labeling, and key on risk comparison scores, controlling for numeracy. The results confirmed our hypotheses about the effect of the logarithmic format and the geometric scale labels, although we did not observe an effect for the integrated versus legend conditions. Specifically, we observed a main effect of format (F_2,1260_ = 4.95, *p =* 0.007), *f* = 0.09 (90% CI: 0.04 – 0.13); see the final row of Table II. However, Tukey's *post hoc* tests, which adjust for multiple comparisons, revealed no statistically significant contrasts.

**Table II:** Experiment 2; Means and Standard Deviations of Risk Comparison Scores (out of 18) by Condition

		Format		
Scale	Key	Standard Matrix	Logarithmic Matrix	Text
1‐5	Legend	7.60 (3.54)	7.40 (3.23)	7.23 (3.66)
	Integrated	7.84 (3.50)	8.36 (3.34)	7.09 (4.11)
1‐625	Legend	10.32 (4.70)	10.10 (4.31)	10.91 (3.90)
	Integrated	10.86 (4.41)	11.05 (4.17)	9.19 (4.25)
Overall		9.11 (4.29)	9.28 (4.05)	8.57 (4.26)

**Fig 7:**
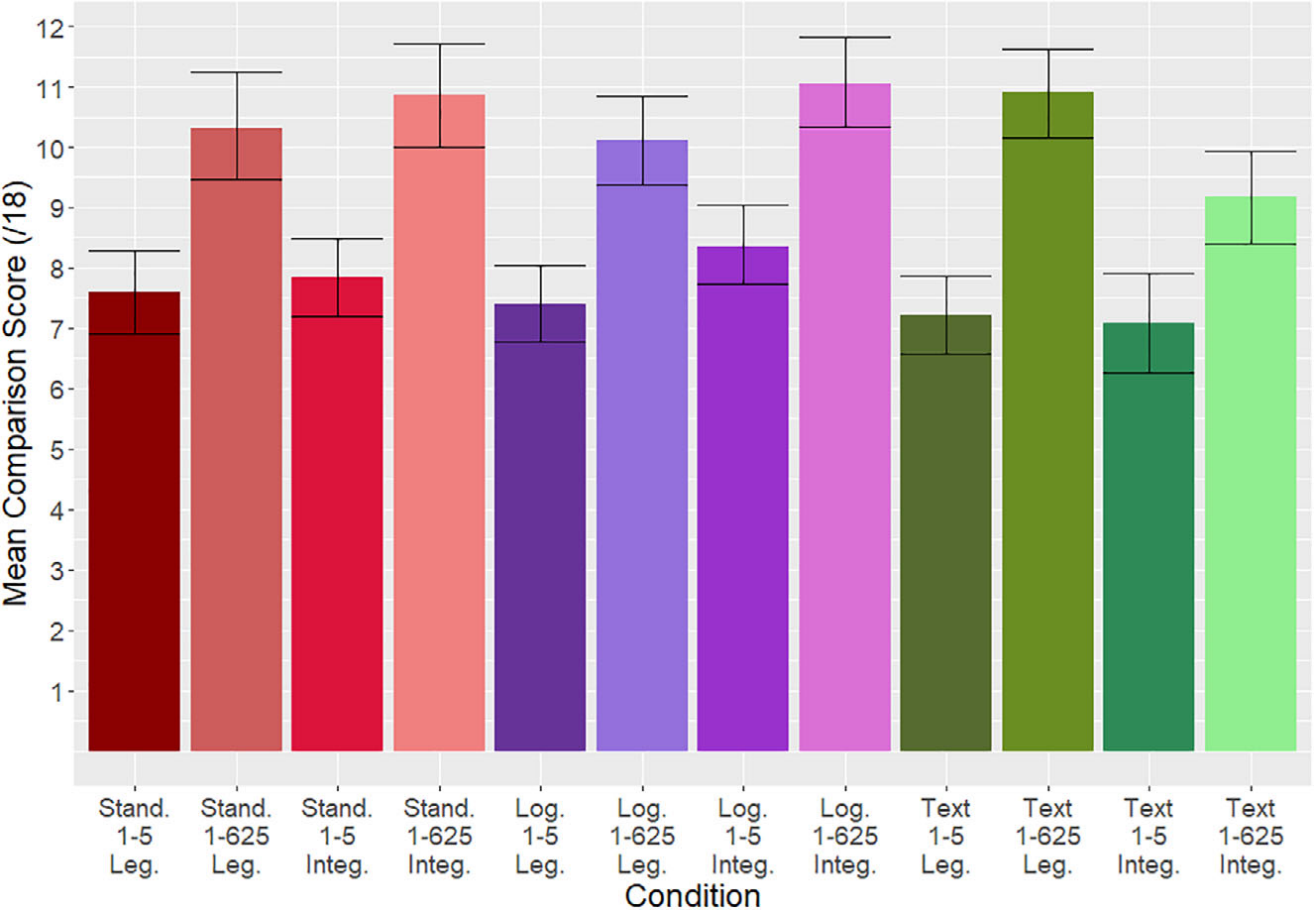
Graph showing the mean risk comparison score per condition, with bootstrapped 95% confidence intervals. Conditions are labeled by format (“Stand.” = standard matrix, “Log.” = logarithmic matrix), scale label, and key (“Leg.” = legends used, “Integ.” = integrated information)

There was also a main effect of scale labeling (F_1,1260_ = 210.54, *p* < 0.0001), with participants who viewed the geometric (1–625) scale labels performing better than those viewing the linear (1–5) scale labels, M(*SD*) 10.39 (4.33) versus 7.61 (3.57) respectively. The effect size of scale labeling on risk comparison score was *f* = 0.41 (90% CI: 0.36 – 0.46). There was no main effect of key (F_1,1260_ = 0.79, *p* = 0.375). Means and standard deviations for every combination of conditions are reported in Table II; see also Fig. 7.

There was additionally an interaction effect of format × key (F_2,1260_ = 10.42, *p* < 0.0001), *f* = 0.13 (90% CI = 0.8−0.17). Descriptively, risk comparison scores were higher with the integrated information (vs. legend) for the standard matrix and logarithmic matrix, but lower for the text (Table III).

**Table III:** Experiment 2; Means and Standard Deviations of Risk Comparison Scores (out of 18), Aggregating over Scale Labeling

	Format		
Key	Standard Matrix	Logarithmic Matrix	Text
Legend	9.02 (4.39)	8.85 (4.07)	8.92 (4.19)
Integrated	9.20 (4.21)	9.68 (3.99)	8.21 (4.30)

Due to concerns that the differences in risk comparison score between the geometric and linear conditions could be primarily driven by a single set of questions within the comparison block (“How many times more likely is risk A than risk B?”; for the linear condition this requires the participant to perform calculations, for the geometric condition this can be read off the axis), the ANCOVA was rerun with those questions excluded from the risk comparison score. The main effect of scale labeling was still highly significant (F_1,1260_ = 146.47, *p* < 0.0001), *f* = 0.34 (90% CI: 0.29 – 0.39). Format, numeracy, and format × key interactions also remained significant at the level of *p* < 0.01. This suggests that improvements in risk comparison score with the geometric scale labels were not solely due to improvements on this single set of questions.

#### 3.2.2 Related Preregistered Analyses: Basic Knowledge Scores and Overall Scores

Because of ceiling effects on the basic knowledge questions, basic knowledge and risk comparison scores for each participant were summed to form an overall performance score (out of 24), to produce a more normal distribution. Overall performance score means and standard deviations for every combination of conditions are reported in Table IV.

**Table IV:** Experiment 2; Means and Standard Deviations of Overall Performance Scores (out of 24) by Condition

		Format		
Scale	Key	Standard Matrix	Logarithmic Matrix	Text
1‐5	Legend	12.48 (4.41)	12.47 (4.02)	11.63 (4.80)
	Integrated	12.77 (4.30)	13.50 (3.98)	11.46 (5.08)
1‐625	Legend	15.11 (5.75)	14.92 (5.47)	15.49 (5.10)
	Integrated	15.74 (5.40)	15.96 (5.02)	13.17 (5.46)
Overall		13.99 (5.16)	14.26 (4.85)	12.90 (5.34)

A three‐way ANCOVA for overall performance score, controlling for numeracy, showed a main effect of format (F_2,1260_ = 13.32, *p* < 0.0001, *f* = 0.15 (90% CI = 0.10−0.19)). A Tukey's *post hoc* revealed that participants viewing the standard and logarithmic matrices performed better than those viewing the text condition (*p* = 0.03 and *p* = 0.003, respectively), though there was no significant difference between the logarithmic and standard matrices (*p* = 0.73); see the final row of Table IV. There was also a main effect of scale labeling (F_1,1260_ = 134.83, *p* < 0.0001, *f* = 0.33 (90% CI = 0.28−0.37)), with participants who viewed the geometric scale labels performing better than those viewing the linear scale labels; means (standard deviations) were 15.06 (5.43) versus 12.42 (4.47), respectively. There was no main effect of key (F_1,1260_ = 0.30, *p* = 0.586).

There was additionally an interaction effect of format × key (F_2,1260_ = 11.61, *p* < 0.0001, *f* = 0.14 (90% CI = 0.9−0.18)). Means in each condition (Table V) exhibited the same descriptive pattern as observed for risk comparison scores (Table III).

**Table V:** Experiment 2; Means and Standard Deviations of Overall Performance Scores (out of 24), Aggregating over Scale Labeling

	Format		
Key	Standard Matrix	Logarithmic Matrix	Text
Legend	13.85 (5.31)	13.79 (4.99)	13.41 (5.29)
Integrated	14.12 (5.04)	14.71 (4.68)	12.37 (5.34)

We did not consider an ANCOVA appropriate for primary analysis of basic knowledge scores due to their severe nonnormality, but we did consider it reasonable as a follow‐up confirmatory analysis to the analysis reported above, given that ANCOVAs are considered fairly robust to normality violation (Olejnik & Algina, 1984), and given that an ANCOVA for basic knowledge (“comprehension”) scores had been preregistered. This analysis is reported in Appendix E section 1.

#### 3.2.3 Other Analyses

As in Experiment 1, we explored whether the different formats affected participants’ overall comprehension and whether this was affected by the amount of previous experience they had with risk matrices. We also considered their subjective preferences between the formats, looked for interactions between format and numeracy, and investigated whether the tendency to select likelihood/impact reduction varied with format. These analyses are reported in Appendix E sections 2 & 3.

### 3.3 Discussion

As hypothesized, the geometric scale labeling (1, 5, 25, 125, 625) substantially improved participants’ ability to answer risk comparison questions, producing the largest effect size observed in this set of experiments (*f* = 0.41). These risk comparison questions required not only understanding of which of two risks was larger, but also the accurate characterization of relative differences in the likelihood and impact between risks, and the use of impact and likelihood information simultaneously to make decisions. A plausible explanation for this improvement is that it made the nonlinear nature of the underlying scales clearer to participants by explicitly showing the fivefold increase in magnitude between adjacent rows or columns—as opposed to the linear labeling (1, 2, 3, 4, 5), where participants had to remember that the underlying scale increased nonlinearly.

As in Experiment 1, in addition to the primary risk comparison measure, a “basic knowledge score” corresponding to the easier measure of comprehension was investigated (reported in Appendix E). One unexpected finding was that participants viewing the geometric scale labeling had lower basic knowledge scores than those viewing the linear scale labeling, although this effect was very weak (*f* = 0.06). Given that the distribution of basic knowledge scores violated ANCOVA assumptions, it is possible that this finding was merely a statistical artefact, although this difference was also found by a nonparametric statistical test. A possible explanation for this discrepancy is that the 1–5 linear scale was more familiar and therefore easier to read, which is relevant given that the basic knowledge questions simply required reading numbers off the diagram. This hypothesis is supported by the fact that the questions driving the difference in basic knowledge scores were of the form, “What is the impact/likelihood of risk A?”. The basic knowledge questions also came before the risk comparison questions, before participants had much practice using the unfamiliar matrix, and may have been differentially affected by its unfamiliarity for this reason.

In any event, participants in the geometric labeling condition performed significantly better on the risk comparison questions, as well as on basic knowledge and risk comparison questions when they were aggregated into a single index (“overall performance”). This was true even when the questions that asked how many times larger one risk was than another were removed, suggesting a global improvement in risk comparison from the geometric labeling. As such, the benefits conveyed by the geometric scale seem to far outweigh the minor dip in performance on the basic knowledge questions, especially if the latter was merely due to an initial lack of familiarity.

Results regarding the integration of information onto the matrix axes/into the text versus the use of a legend were equivocal. There was a small interaction effect (*f* = 0.13) suggesting that integrated information improved performance for both matrix conditions, but worsened it for the text condition. This may be due to an “information overload” effect with the text condition: integrating the information made the sentences much longer and denser than with the legend, and the resulting “cognitive load” (Paas, Renkl, & Sweller, 2004) may have made it more difficult to identify key pieces of information. It may also be that the loss of some information in the integrated text condition contributed to the poorer performance: Participants in this condition were not shown the full range of possible values of likelihood and impact scales, unlike participants in both the integrated and legend matrix conditions, due to the inherent difficulty of adapting conditions that were designed for use with a matrix to text stimuli. In short, the way in which information was integrated in the integrated text condition was necessarily fundamentally different from the way in which it was integrated into the visual matrices, so it is perhaps unsurprising to find an interaction with no main effect. Finally, as in Experiment 1, we also saw an overall preference to reduce impact over likelihood, which was not affected by condition.

## 4 GENERAL DISCUSSION

In this pair of experiments we set out to investigate the effects of risk matrix cell format and axis labeling on a public audience's comprehension and judgment. We were particularly interested in how to help people compare risks positioned relative to each other on nonlinear axes.

The results from both experiments in the study show that, across all measures, text alone generally performed worse than at least one of the matrix conditions, suggesting that there may be an advantage in using some kinds of risk matrices to represent risk likelihood and impact. The effect sizes were generally very small, however, perhaps because in this study only one or two risks were illustrated per matrix, meaning that the information presented was relatively simple.

Nevertheless, out of the three matrix cell designs we tested (square, rectangular and “logarithmic”), Experiment 1 found that the “logarithmic” format alone resulted in a small but significant benefit as compared with text. In Experiment 2, we found a main effect of format with the same descriptive pattern of means (text < standard < logarithmic) on the measure used in Experiment 1, and found advantages for the logarithmic and standard matrices vs. text when using a more comprehensive “overall performance” measure. A more robust finding was that a geometric labeling (in this experiment, a geometric scale with labels of 1, 5, 25, 125, and 625) created an improvement in scores of moderate effect size (*f* = 0.41 for risk comparison, *f* = 0.33 for overall performance), suggesting that emphasizing a nonlinear scale with nonlinear labeling is more salient than attempting to do so with the shape of the matrix cells. We also found some evidence that, for matrices, integrating quantitative information about the likelihood and impact scales onto the axes may have helped people take it into account more effectively than when it was consigned to a separate key.

When combined with relevant theory, some of these experimental results offer hints about possible mechanisms behind some of the differences observed, which may inform the design of a wider range of risk communication visualizations than the specific subset analyzed here. In addition to the possible effects of cognitive load, potential mechanisms include effects on top‐down knowledge and bottom‐up processing as well as familiarity effects.

### 4.1 The Possible Role of Top‐Down Knowledge and Bottom‐up Processing

As discussed above, in Experiment 1, the use of a “logarithmic” cell design improved comprehension of the nonlinear nature of matrix axes above that of a text‐based control. This result could be attributable simply to bottom‐up processing, where the “logarithmic” cell design works with (rather than against) cognitive heuristics such as magnitude priming. In other words, although such heuristics can be subject to bias and result in irrational decision making (Tversky & Kahneman, 1974), when applied in the right circumstances they can result in more “rational” judgements and decisions than a more deliberative, top‐down process might (Gigerenzer & Brighton, 2009). Alternatively, and concordant with the presumed importance of top‐down knowledge for the results observed by Heckler et al. (2013), who found that labeling log scales using tick marks with varied spacing improved comprehension, such an effect could be explained via an interaction between bottom‐up processing and top‐down knowledge. Specifically, the increasing size of cells toward the top right of the “logarithmic” matrix may have provided a bottom‐up visual cue to users that in turn reminded them of the nonlinear nature of the underlying scale (top‐down knowledge), thus facilitating better comprehension of the nonlinear nature of the matrix axes. Of course, both processes could have been at work independently; there is evidence for the importance of both domain‐specific and domain‐general top‐down knowledge in graph comprehension (Pinker, 1990; Shah & Freedman, 2011).

### 4.2 Familiarity Effects

Across the two experiments, we also saw effects that are likely due to familiarity or unfamiliarity with formats. For example, the comparatively high performance with the standard matrix among participants who said that they were used to risk matrices (see Appendices) is likely because matrix‐familiar participants already have training in that particular format and are therefore comfortable using it; whereas, for the logarithmic matrix and text condition, they have to first learn a new graphic schema (Pinker, 1990). Similarly, participants generally expressed a subjective preference for the format they had seen during the experiment—the well‐known “mere exposure” effect (Zajonc, 1968)—and a relative aversion to “unusual” formats, such as the logarithmic matrix and geometrically increasing scale labels. The latter are likely unfamiliar within a broader context of graphical formats experienced in everyday life, where table cells are generally evenly sized, and scales are generally arithmetically rather than geometrically increasing.

Participants’ preference of familiar over unfamiliar formats may raise concerns for some risk managers who would otherwise consider altering their risk matrix format in light of our findings on comprehension and performance. Several facts should ease such qualms. First, while the preference differences between conditions were significant, they hardly suggest a burning hatred for novel formats. Among all participants, 42% preferred the linear scale labels, 35% preferred the geometric scale labels, and 22% had no preference; similarly, 42% preferred the standard matrix, 30% the logarithmic matrix, 13% the text, and 15% no preference (Appendix E). Additionally, confidence intervals around proportions of participants viewing each format suggested that the geometric labels—the change that had the greatest positive effect on risk comparison scores—were as likely to be preferred as the linear labels by those who were randomized to use the geometric scale labels for the brief duration of the experiment. This is presumably due to the “mere exposure” effect, but also potentially because participants noticed that they made the task easier than it might have otherwise been. This is good news, as it suggests that there is little downside to the use of geometrically increasing scale labels, other than the fact that new users may initially prefer linear scale labels until they spend a few minutes building up some familiarity with the new format. Note also that preferences for graphical formats of risk communications are not generally associated with how well individuals understand the risks being communicated (Barnes, Hanoch, Miron‐Shatz, & Ozanne, 2016; Hamstra et al., 2015; van Weert, Alblas, van Dijk, & Jansen, 2021).

Our recommendations in the Conclusion are therefore more closely guided by our findings on comprehension than they are by preferences and self‐reported ratings. One caveat is that matrix‐familiar participants may have a different pattern of performance than matrix unfamiliar participants. This effect was not caused by differing numeracy scores between the two groups—for both experiments, there was only a small difference between numeracy scores for matrix‐familiar and matrix‐unfamiliar participants. It seems likely to be an effect of familiarity, or perhaps even a statistical artefact given matrix‐familiar participants made up a small proportion of the overall sample (359 out of 1,260). Further work on this specific population is needed, and risk managers considering making changes to risk matrices for an audience with prior risk matrix experience may wish to carry out familiarization and training before introducing a new format of any kind.

### 4.3 Limitations

This study only examined semiqualitative matrices. Though qualitative and quantitative matrices may share some design concerns with semiqualitative matrices, they have their own specific concerns that cannot be covered here. There are also broader issues regarding their methodology and, in the case of quantitative matrices, their underlying calculations that perhaps outweigh any design concerns. See Monat & Doremus (2018) for a further account of some mathematical issues with quantitative matrices.

Additionally, it is possible that we missed effects that we were theoretically powered to detect due to the reduction in power resulting from the use of alternative tests when ANOVA assumptions were violated, for example, the partial proportional odds model and the Mann–Whitney *U* test. This risk was, however, mitigated by running confirmatory ANOVAs (which are relatively robust to normality violations) in instances where nonnormality resulted in us choosing a nonparametric test for the primary analysis (see relevant appendices for more details). It is also possible that some effects were not detected by some tests run on subgroups of the participant population, for example, people who had prior experience with risk matrices, as this subsampling similarly resulted in a reduction in power.

Finally, this study tested a small space within the broad range of risk matrix formats and options available. A search of matrices found in various academic papers, in governmental and industry reports, and online shows an enormous amount of variation in design. Other improvements are likely to be discovered by further exploration of formats.

### 4.4 Ethical Use of Risk Matrices

Many organizations have an ethical imperative to make decisions that reduce risks to life, health, and property, and to ensure that these decisions are based on sound judgment and accurate impressions of the likelihood and consequence of these risks. We hope that the recommendations and findings here will facilitate these aims. However, many important factors are not represented in risk matrices, and there are ethical problems with relying upon them in an overly narrow fashion. For example, all decisions necessarily have positive and negative externalities that are not included in the measure of “impact” used by the risk assessment, which must also be taken into account. Some have also argued that the use of numbers in difficult‐to‐quantify risk assessments is misleading. For example, the specific numbers on our geometric scale (1, 5, 25, 125, 625) could conceivably cause an imprecise risk assessment to be interpreted as more precise than it truly is. While it is our contention that the “wiggle room” afforded by imprecise qualitative language leads to far greater ethical risk—for example, the notoriously variable interpretations that intelligence analysts ascribe to phrases like “serious possibility,” ranging from odds of 80/20 to 20/80 (Tetlock & Gardner, 2015)—the appropriate communication of uncertainty in risk estimates is extremely important. It is crucial that risk matrices not be used as a replacement for critical thinking, but rather as one tool in a toolbox for ethical decision making.

### 4.5 Future Directions

Many risk matrices use color in their cells to indicate severity of risk, acceptability/tolerance of risk, or prioritization of remediation. Concerns have been raised previously, notably in Monat and Doremus (2018), that these can affect judgements in unintended ways. Experimental work in this area would be useful to examine for such effects. Other aspects of risk matrices that require investigation include how risks are presented on the matrix, how changes to risks (e.g., difference between impact/likelihood of a risk before and after mitigation) are presented, the benefits and drawbacks of continuous versus categorical representations, and how the uncertainty of risk estimates is represented.

## 5 CONCLUSIONS

The current study suggests that there are changes to the standard format of qualitative and semiqualitative risk matrices (rectangular cells, linear scale labeling, use of a key) that may help them to communicate risk more effectively. Based on evidence from Experiment 2, the primary recommendation for an improved risk presentation format is the use of ordinal, explicitly nonlinear scale labels for matrices with an exponential or otherwise nonlinear increase in likelihood and/or impact along the axes. These types of scales represent the nonlinear change from one cell to another by increasing in a suitable geometric progression (e.g., 1, 5, 25, 125, 625).

Evidence from Experiments 1 and 2 also suggests a possible benefit of a “logarithmic” format with increasing spacing between lines, although it should be emphasized that this may not be true for people who are already familiar with risk matrices, and that significant differences between the logarithmic and standard formats were not observed in either study.

There was no statistically significant main effect of integrating information about the meaning of each column and row into the display versus placing it in a separate legend. However, interactions suggested that integrated information may have improved task performance for the standard and logarithmic matrices, but worsened task performance for the text conditions. This finding is supported by prior research regarding the benefits of integrating information into diagrams wherever possible (Carpenter & Shah, 1998). It may be worth considering integrating any information currently in a key onto the matrix, where this is graphically reasonable.

**Fig 8:**
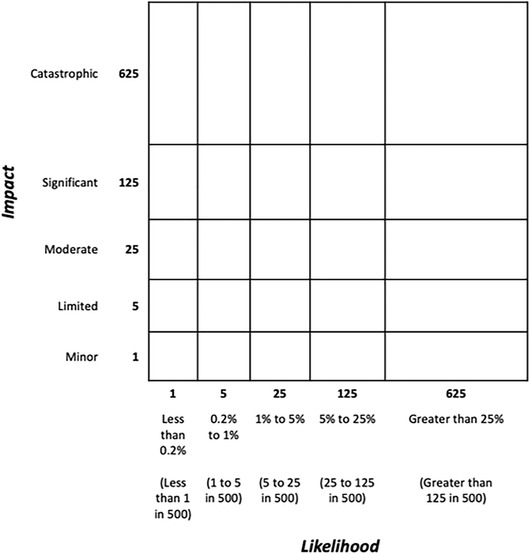
A matrix showing the three features recommended as a result of this study: geometrically increasing labels for each axis, representing the nonlinear nature of the underlying scale; increasing cell sizes to reinforce the nonlinear nature of the scale; and information about likelihood and/or impact integrated directly into the matrix (rather than in a separate legend) for ease of reference

Some of these changes (logarithmic matrix; integrated information) may not be subjectively preferred by users on first experience despite their benefits, but opinions are likely to improve swiftly with familiarity. The change with most consistent beneficial effect (geometric scale labeling) was not dispreferred by participants who had been randomized to use matrices with this labeling over the course of the experiment. See Fig. 8 for an example of our recommended matrix format.

More broadly, we would recommend the use of cues that remind users of the true scale of the axes, and which minimize cognitive load, even if these changes are met with some initial resistance. If our findings in Experiment 2 are any guide, users are likely to feel much more comfortable and confident with the new display after a short amount of practice and regular use.

6

## Supporting information

Supplementary material

## Data Availability

Data, stimuli, and the questionnaires from the two experiments is freely available at https://osf.io/6fnu2/?view_only=0a09e85f5fd24e4ea88e16f9f515c416.

## Footnotes

1 For example, the physical distance between the centers (or left edges) of cells 1 and 2 would be smaller than the distance between the centers (or left edges) of cells 4 and 5.

2 Standards differ by industry with respect to whether likelihood or impact is represented on the *y*‐axis. Generally, the longest side of the matrix is whichever side is against the *y*‐axis, though there is no real reason that this need be the case.

3 For example, “What is the likelihood/impact of A?” is a question set, made up of multiple questions with different stimuli. See Supporting Information Figs. G1–G2.
