# Supplementary material for: How People Understand Risk Matrices, and How Matrix Design Can Improve their Use: Findings from Randomized Controlled Studies
Source: Risk Anal. 2021 Sep 14;42(5):1023–41. doi: 10.1111/risa.13822 (PMC9544625; doi:10.1111/risa.13822)

**APPENDICES**

[**APPENDIX A. Experiment 1 Instructions** 35](#_Toc80623002)

[**APPENDIX B. Deviations from pre-registrations** 37](#_Toc80623003)

[**APPENDIX C. Experiment 1 – Other Analyses** 39](#_Toc80623004)

[**APPENDIX D. Experiment 2 Stimuli** 42](#_Toc80623005)

[**APPENDIX E. Experiment 2 – Other Analyses** 47](#_Toc80623006)

[**APPENDIX F. Supplementary Tables** 55](#_Toc80623007)

[**APPENDIX G. Supplementary Figures** 65](#_Toc80623008)

# **APPENDIX A. Experiment 1 Instructions**

Please note that the stimuli shown here are smaller than they appeared in the experiment.

**Instructions for matrix stimuli:**

Information about risks can be communicated in many different ways. One possible risk communication format is shown above.

Along the bottom of the grid is the likelihood of a risk - “How likely is this risk to happen?”. This is shown on a 1 to 5 scale, where each increase of 1 on the scale means the likelihood is five times greater. The chances of an event happening, for each level of the scale, are shown in the Likelihood Key below the grid. They are shown both as a percentage and as a frequency.

Up the left side of the grid is the potential impact of a risk - “If this risk happens, how bad will it be?”.  This is provided using a 1 to 5 scale, where each increase of 1 on the scale means the potential impact is five times worse. The descriptive labels for each level of the scale are shown in the Impact Key to the left of the grid.

By putting a risk in a particular place on the grid (here, shown by A in the diagram above, to stand for e.g. the risk of a flood in Town A), we can communicate both the likelihood and impact of that risk at the same time.

**Further instructions:**

This risk presentation format can also be used to show a change in risk (or a change in two risks), as shown above. Risk A on the left is the initial risk (e.g. the risk of a flood in Town A), and risk A on the right is the risk after something has changed about the situation (e.g. Town A has put up flood barriers).

**Instructions for text stimulus:**

Information about risks can be communicated in many different ways. One possible risk communication format is shown above.

The text provides information about the **likelihood** of a risk - “How likely is this risk to happen?”. This is provided using a 1 to 5 scale, where**each increase of 1 on the scale means the likelihood is five times greater.** The chances of an event happening, for each level of the scale, are shown in the Likelihood Key below the text. They are shown both as a percentage and as a frequency.
 
The text also provides information about the **potential impact**of a risk - “If this risk happens, how bad will it be?”. This is provided using a 1 to 5 scale, where **each increase of 1 on the scale means the potential impact is five times worse**. The descriptive labels for each level of the scale are shown in the Impact Key to the left of the text.

# **APPENDIX B. Deviations from pre-registrations**

Pre-registrations referred to what this manuscript refers to as “basic knowledge” questions as “comprehension” questions. This measure was renamed to “basic knowledge” for the manuscript to avoid confusion with the broader concept of comprehension, which includes both the understanding of how likelihoods and impacts are represented on the matrix (basic knowledge) and how to accurately compare multiple risks in a way that demonstrates understanding the non-linear nature of the matrix’s axes (risk comparison questions). Free text response boxes after each impact vs. likelihood question were removed prior to running the pilot, as they added too much to the survey length. An ANCOVA for the effect of condition on risk comparison score was pre-registered as an experimental analysis for Experiment 1. However, due to significant violations of homogeneity of variance a partial proportional odds ratio model was used instead. An ANCOVA was also pre-registered to assess the likelihood vs. impact questions for impact bias. However, this was not sufficient as the sole analysis and a t-test was added. An ANCOVA for the effect of condition on basic knowledge score was pre-registered as an experimental analysis. However, this was not appropriate due to the strong ceiling effect seen in basic knowledge performance across all arms, which resulted in a strongly non-normal distribution. Instead, basic knowledge and risk comparison scores were combined to create a more normally-distributed ‘overall performance score’, which represented the participant’s ability to understand and successfully carry out both simple (basic knowledge score) and more complex (risk comparison score) operations using the matrix. This overall performance score was then used for the ANCOVA instead.

The pre-registration for Experiment 2 suggested that the one-sample t-test would compare bias average to a value of 0.5, which is incorrect – given the coding of likelihood and impact responses, the comparison value should be 0.

As the original paper validating the Berlin Numeracy Test recommended it be combined with “at least one other test” of numeracy for general population samples to reduce skew and provide better discrimination between low-numeracy participants (Cokely et al., 2012), an aggregate numeracy measure was used as described in section 2.1.3 of the manuscript and in our public preregistration for Experiment 2. We note that all significant findings in the main manuscript remain significant, and no non-significant findings become significant, if the item from the Expanded Numeracy Scale is excluded, or if only the Berlin is used.

# **APPENDIX C. Experiment 1 – Other Analyses**

**C1. Participants with Prior Risk Matrix Experience**

The 404 participants who reported some experience with using risk matrices (“yes, rarely”, “yes, sometimes”, and “yes, often”) were analysed as a subset, to assess whether any patterns of performance change were different in this subset than in the participant cohort as a whole. Means and standard deviations for this subset are reported in Supplementary Table F2. An ANCOVA conducted on this subset to determine the effect of format on risk comparison score, controlling for numeracy, showed no significant effect of format (F_3,399_=2.22, *p*=0.085). Repeating the analysis with overall score as the dependent variable also showed no significant effect of format (F_3,399_=1.98, *p*=0.117). ANCOVAs were used here as this subset did not violate the homogeneity of variance assumption. These results, however, should be interpreted with caution, due to reduced statistical power.

**C2. Matrix Preference**

As described in the Methods, after completing tasks using the matrix format corresponding to their format condition, participants were presented with all formats and asked to indicate which they preferred. A chi-squared test of independence was performed to examine the relationship between format seen during tasks and format selected. The relationship between these variables was significant *X^2^*(12, *N*=1426) =191.72, *p*<0.0001. Exact numbers of participants preferring each format in each condition are reported in Supplementary Table F3.

Participants shown the standard matrix were most likely to prefer the standard matrix (est.=37%, 95% CI=32%–43%). Of participants shown the standard matrix, both standard and square matrices were more likely to be preferred over plain text and ‘none of the above’ (i.e., confidence intervals did not overlap with 32%-43%).

Participants shown the square matrix were more likely to prefer the square matrix (est.=47%, 95% CI=42%–52%) than any other format. Participants who saw the logarithmic matrix were most likely to prefer the logarithmic matrix (est.=33%, 95% CI: 27% – 38%) or the square matrix (est.=35%, 95% CI: 30%– 40%) over the other format options.

Finally, participants shown plain text were more likely to prefer plain text (est.=28%, 95% CI: 23% – 34%) over ‘none of the above’ (est.=13%, 95% CI: 8% – 18%); the proportion of these participants who preferred the other options was similar (confidence intervals overlapped), indicating a lack of clear preference.

**C3. Subjective Feedback**

For graphs of pre-task and post-task ratings with means, please see Appendix D. ANCOVAs showed no significant effect of format on participants’ pre-task comprehension ratings (F_3,1421_=0.59, *p*=0.622), post-task comprehension ratings (F_3,1421_=0.55, *p*=0.646), pre-task ease of use ratings (F_3,1421_=0.45, *p*=0.715), post-task ease of use rating (F_3,1421_=1.32, *p*=0.267), difference between pre- and post-task comprehension (F_3,1421_=0.72, *p*=0.538), difference between pre- and post-task ease of use (F_3,1421_=2.11, *p*=0.098), post-task ‘confidence using format oneself’ rating (F_3,1421_=1.07, *p*=0.357) or post-task ‘confidence explaining format to others’ rating (F_3,1421_=1.39, *p*=0.244), controlling for the effects of numeracy in each case.

Subjective preferences showed a clear effect of familiarity, with many participants preferring the format that they had already been shown. However, the square matrix was also the favourite of many participants who had been shown other formats. Given the lack of evidence for improved performance with the square matrix over the standard matrix and text format, and given that the square matrix is not the most common type of matrix used generally, this finding is unexpected and hard to explain. For participants who had been shown only plain text, there was no clear evidence that text was more likely to be preferred over any other format, perhaps indicating that visual representations of the data were perceived as easier to parse than text descriptions.

**C4. Interactions with Numeracy**

An exploratory repeat of the analysis in section 2.2.1 of the main manuscript, but in which format×numeracy interaction terms were included in the model (format_standard_×numeracy, format_square_×numeracy, format_log_×numeracy) revealed significant interactions for format_standard_×numeracy (est.=-0.17, *p*=.009) and format_log_×numeracy (est.=-0.12, *p*=0.047). Visualizing these suggested that the slope of the line relating numeracy and risk comparison scores was flatter for the standard and log matrices than for the text control (Supplementary Fig. G3).

# **APPENDIX D. Experiment 2 Stimuli**

Please note that the stimuli shown here are smaller than they appeared in the experiment.

Standard matrix; linear scale labelling; legend:


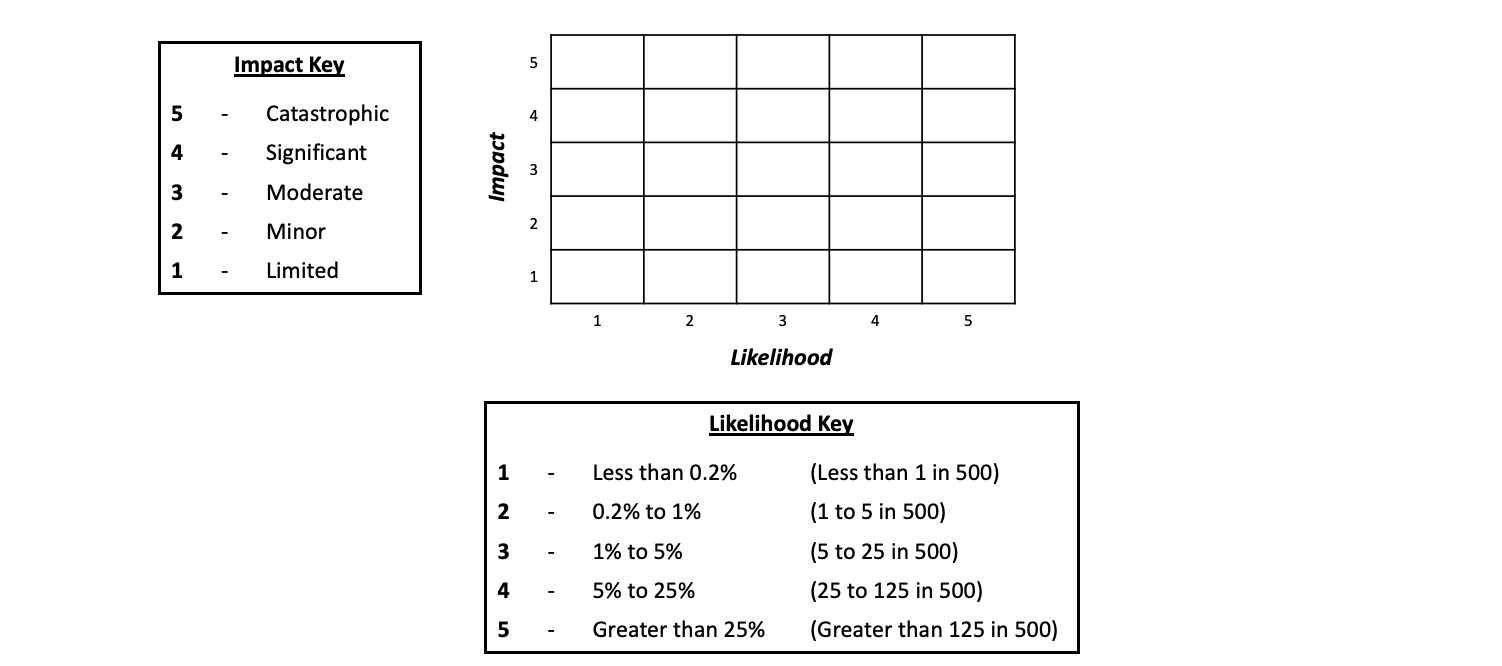


Standard matrix; geometric scale labelling; legend:


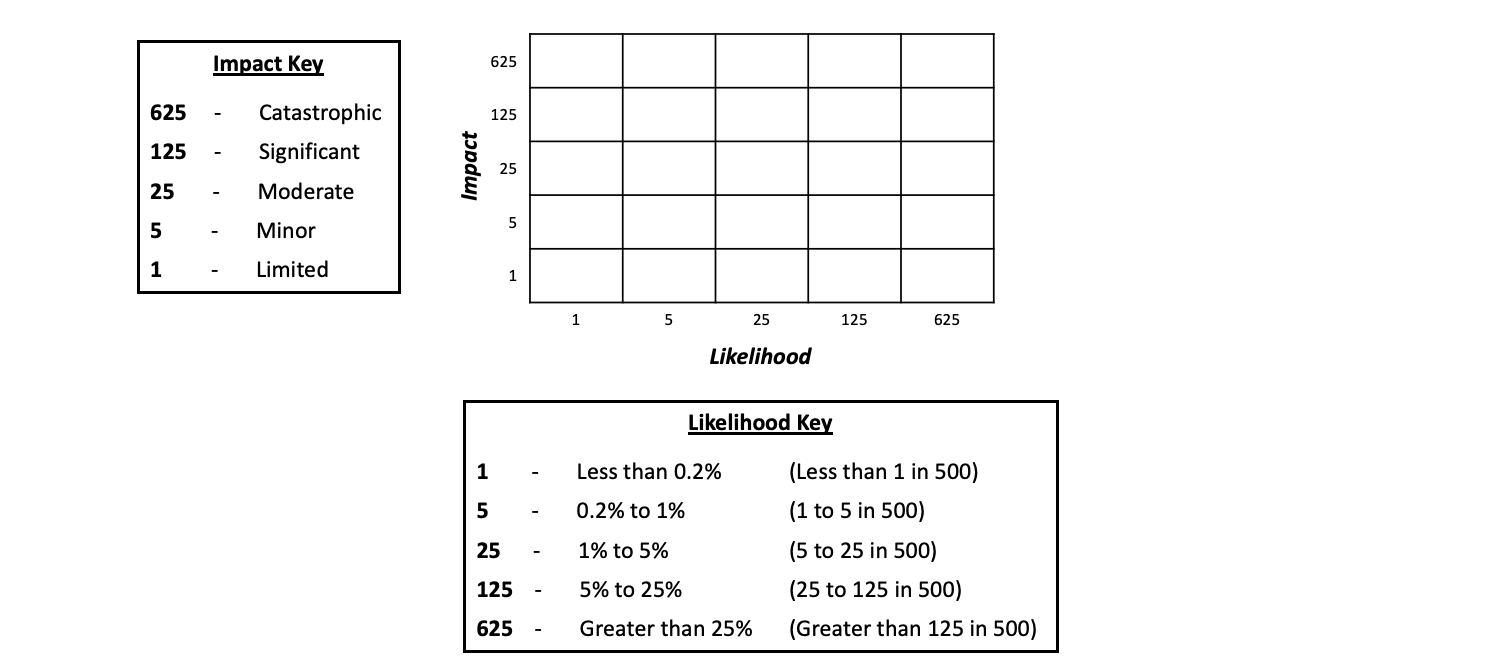


Standard matrix; linear scale labelling; integrated information:


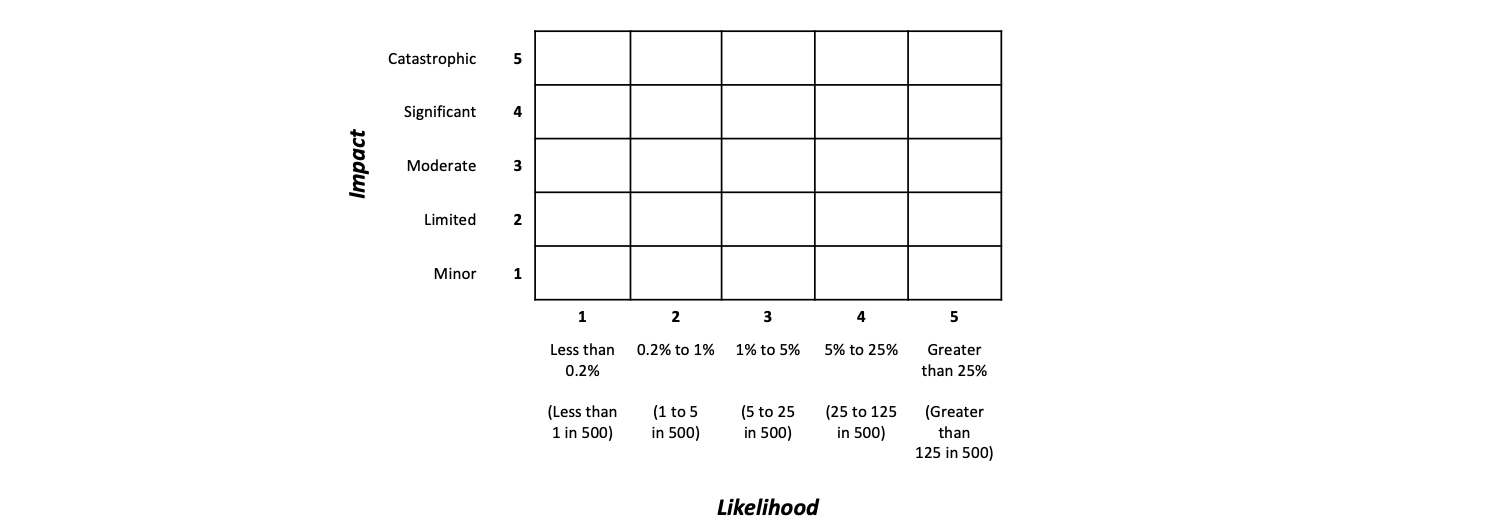


Standard matrix: geometric scale labelling; integrated information:


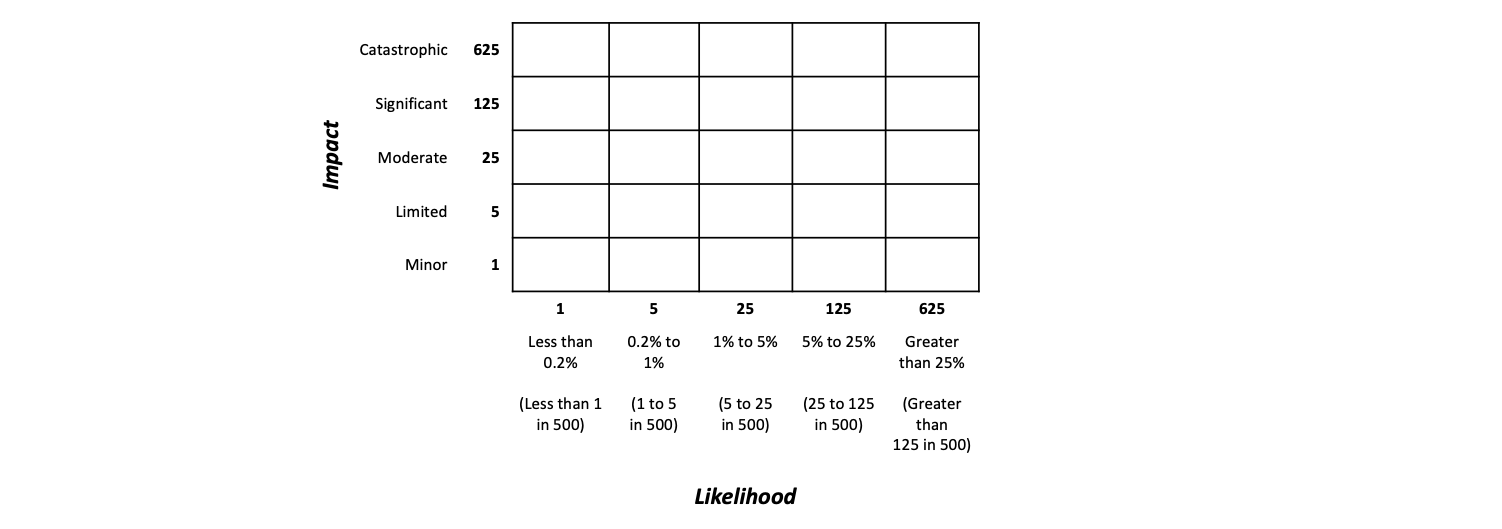


Log matrix; linear scale labelling; legend:


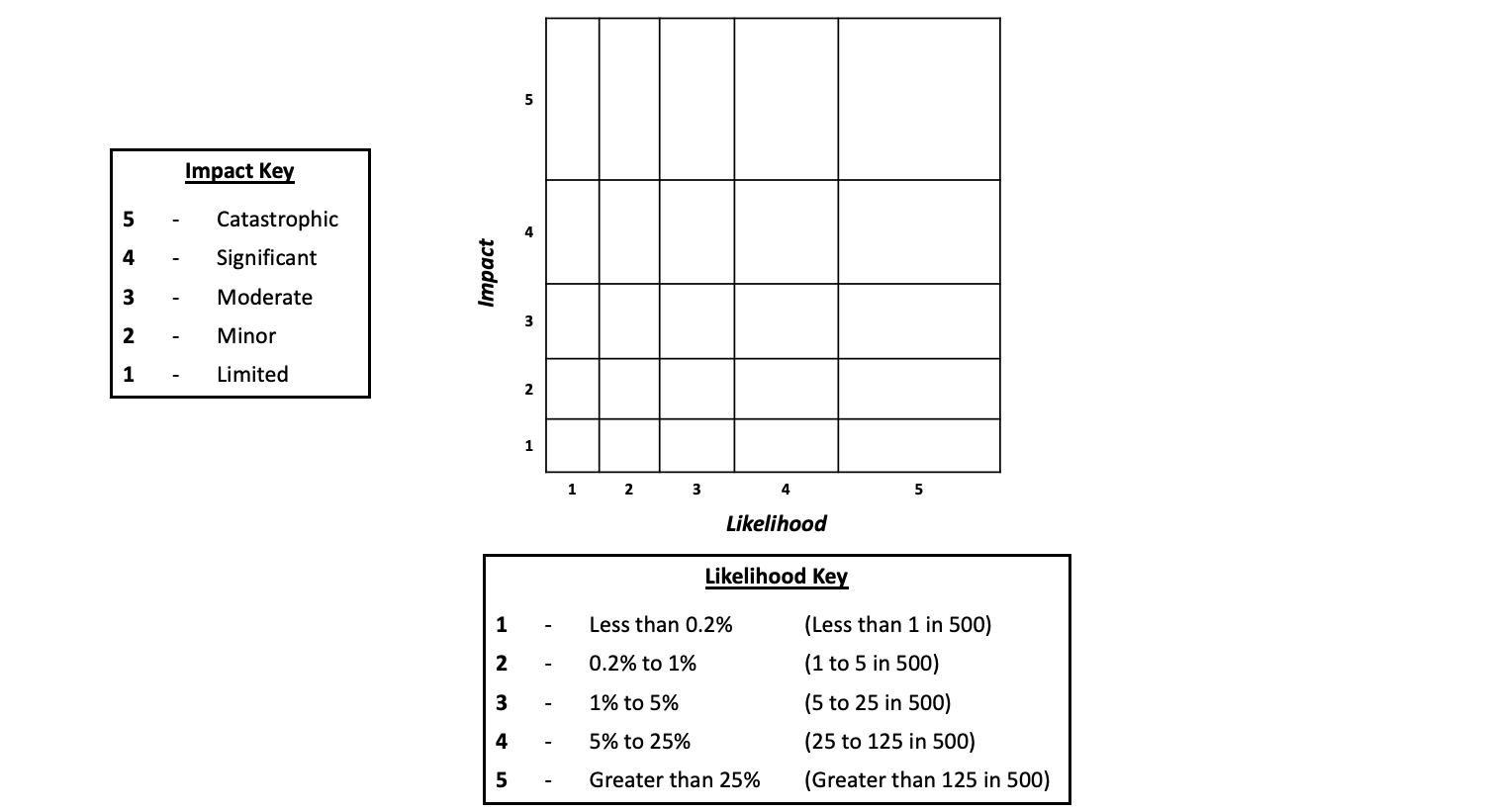


Log matrix; geometric scale labelling; legend:


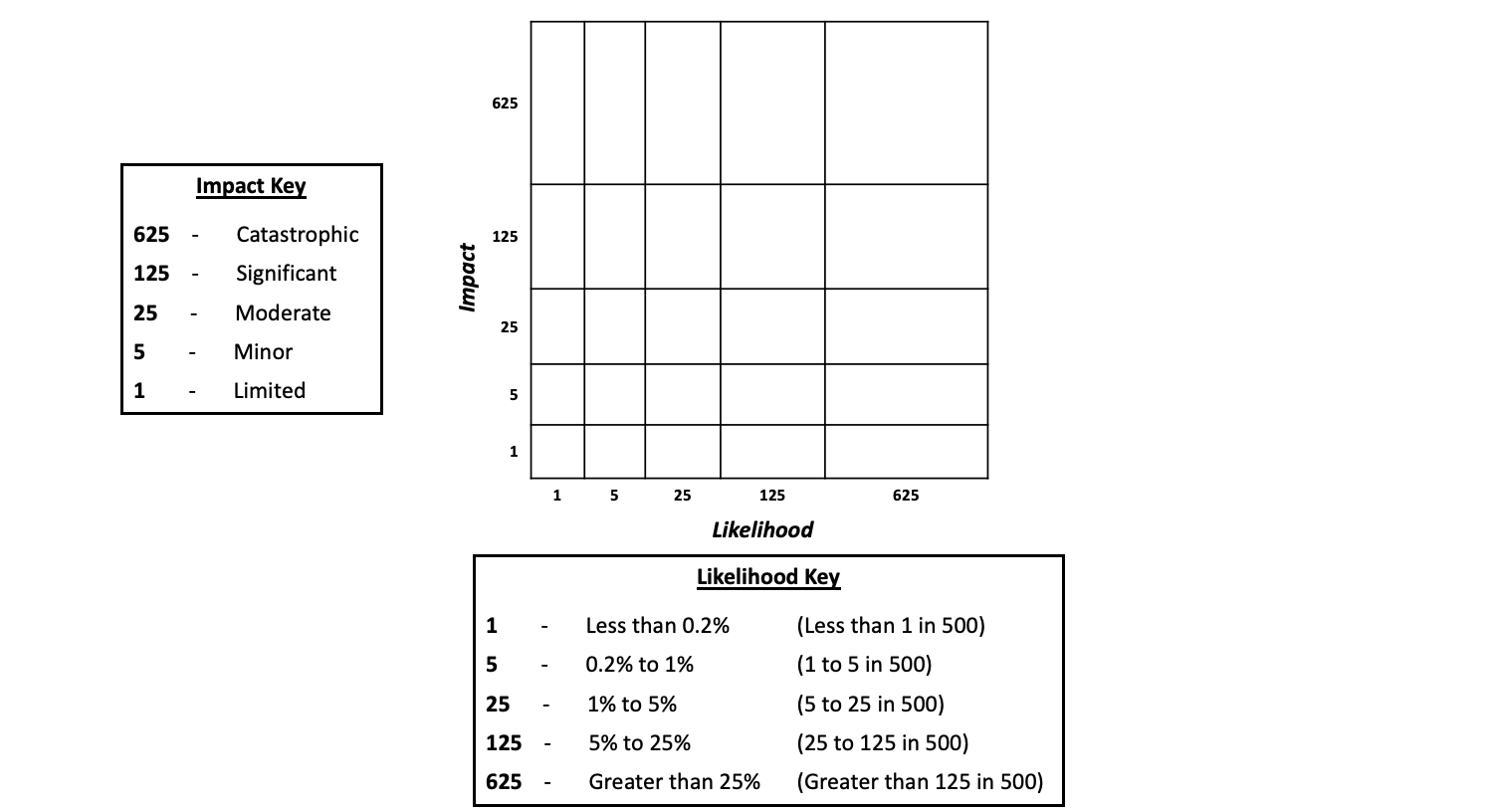


Log matrix; linear scale labelling; integrated information:


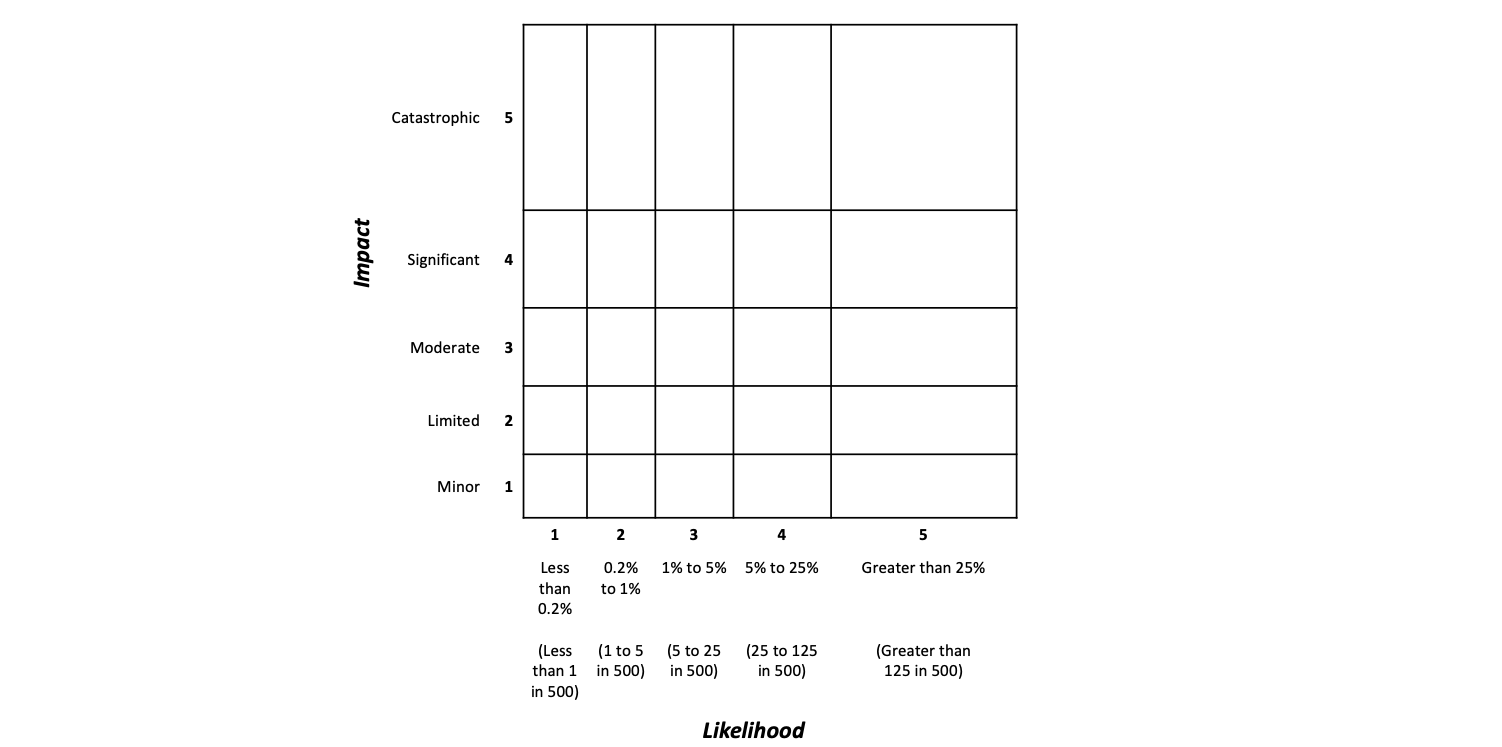


Log matrix: geometric scale labelling; integrated information:


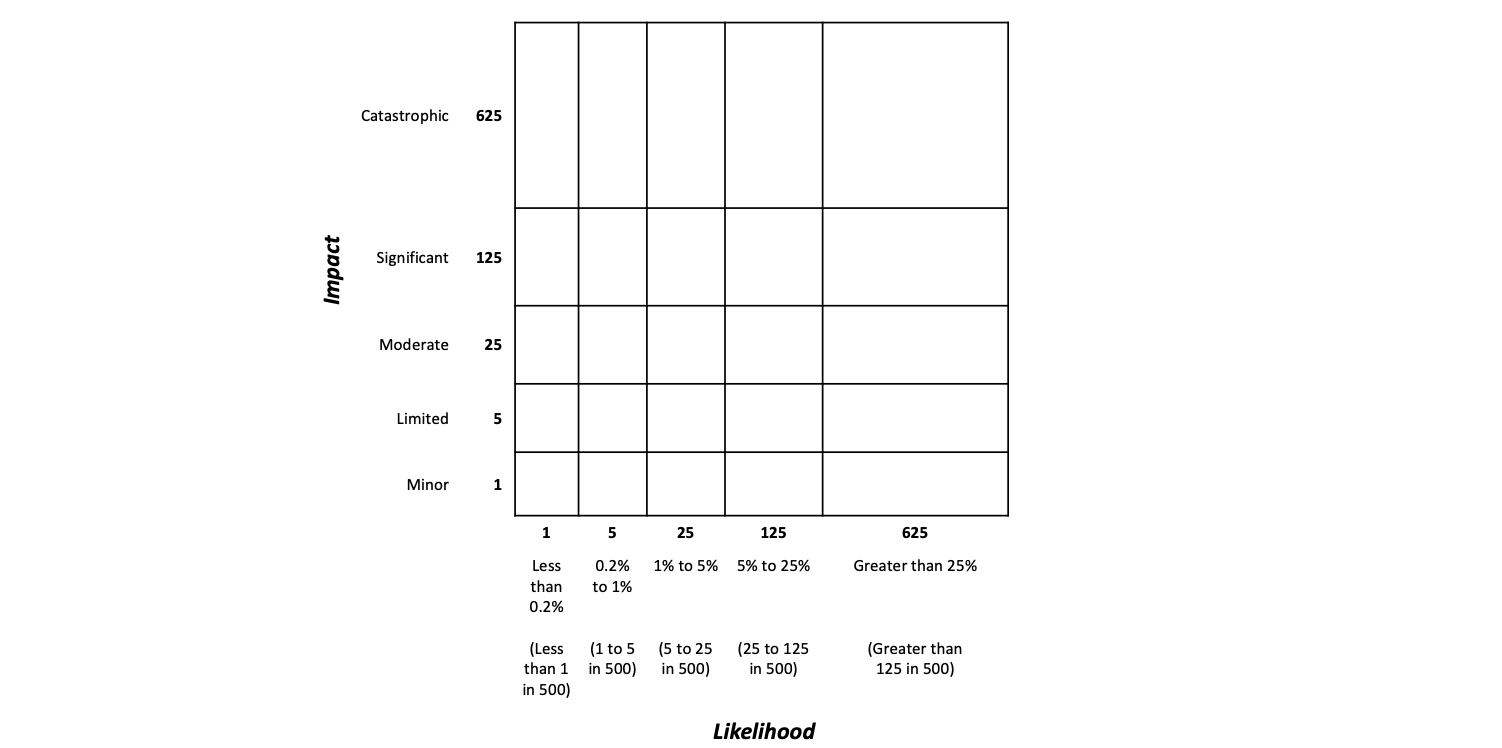


Text; linear scale labelling; legend:


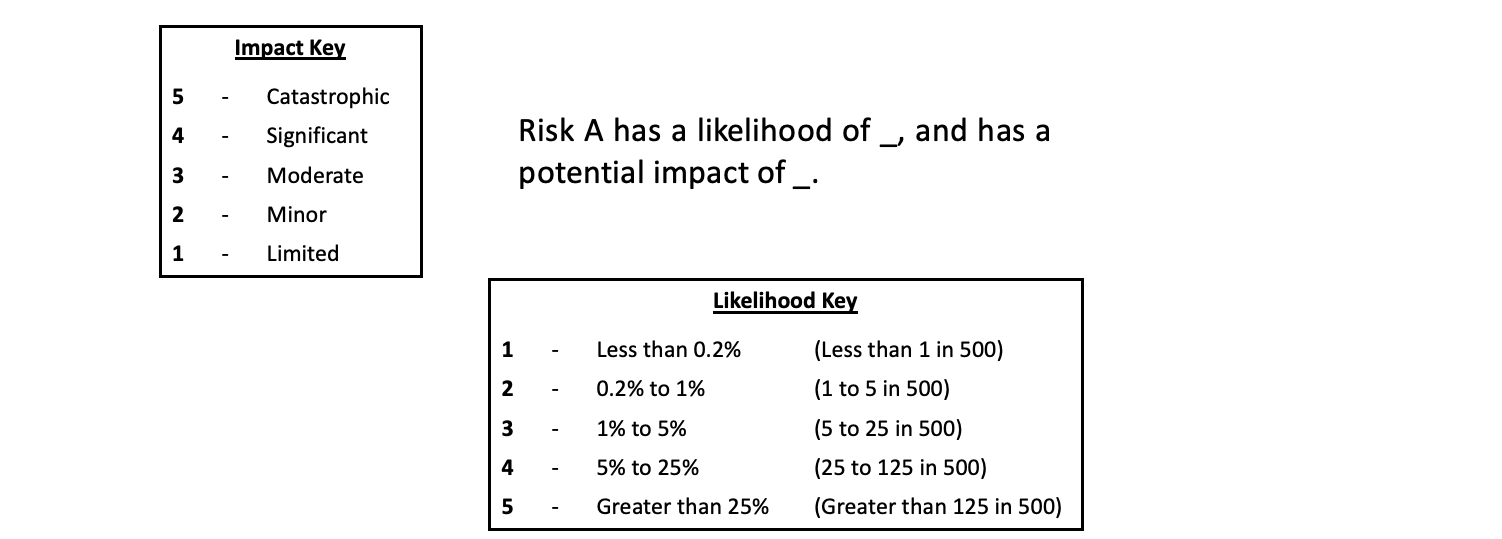


Text; geometric scale labelling; legend:


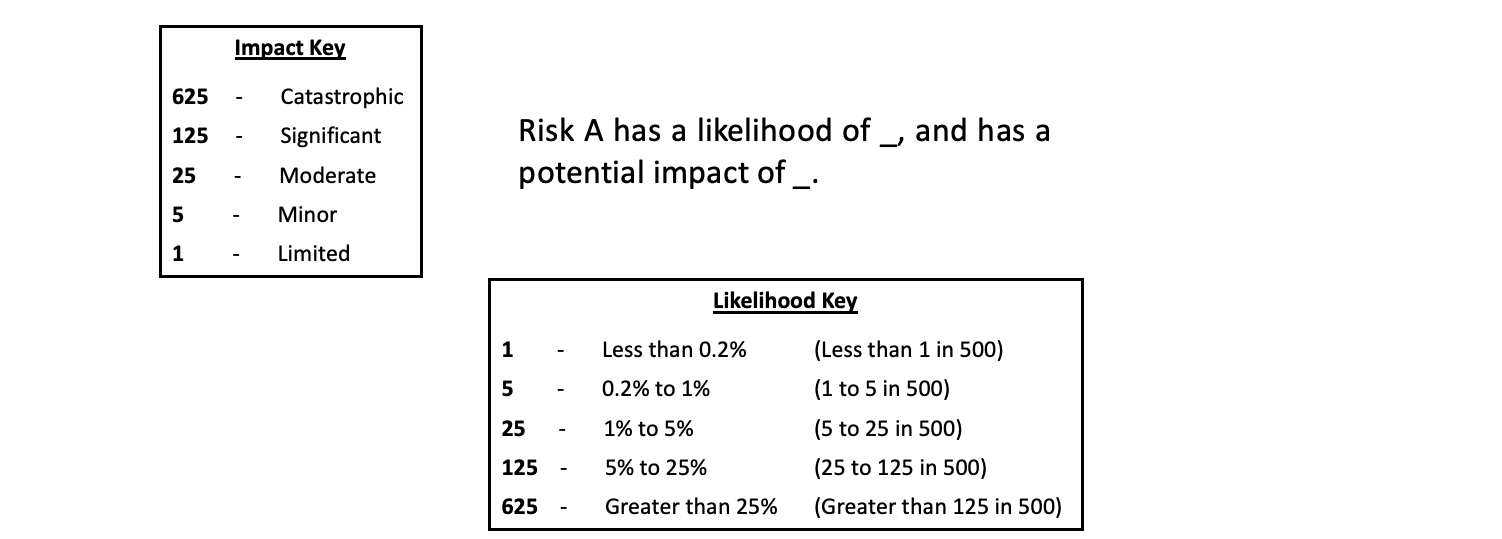


Text; linear scale labelling; integrated information:


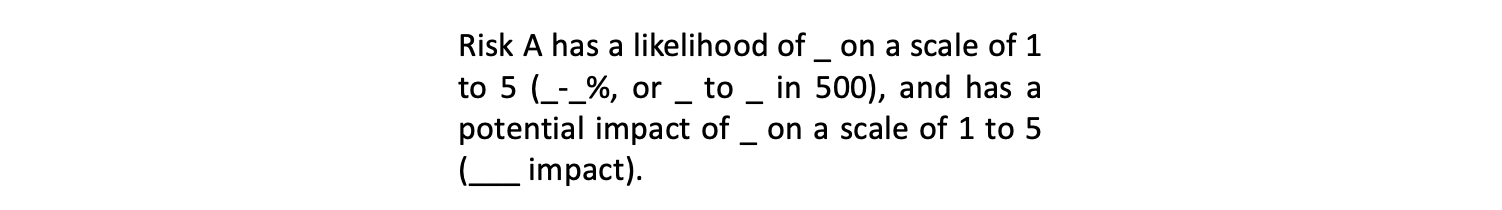


Text: geometric scale labelling; integrated information:

**
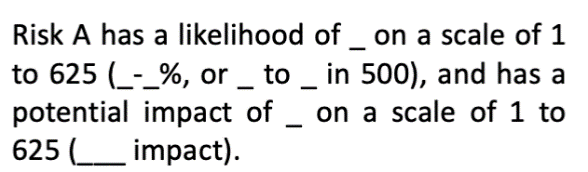
**

# **APPENDIX E. Experiment 2 – Other Analyses**

**E1. Confirmatory ANCOVA for Basic Knowledge Scores**

A confirmatory three-way ANCOVA was conducted to determine the effect of format by scale labelling by key on basic knowledge score, controlling for numeracy (by using participants’ total numeracy score as a covariate). As with the overall score ANCOVA, there were main effects of format (F_2,1260_=29.35, *p*<0.0001, *f*=0.22 (90% CI=0.17–0.26)) and of scale labelling (F_1,2196_=4.023, *p*=0.045, *f*=0.06 (90% CI=0.01–0.10)), but not of key (F_1,2196_=0.39, *p*=0.530), with an interaction effect of format×key (F_2,1260_=4.50, *p*=0.011, *f*=0.08 (95% CI=0.03–0.13)). See Supplementary Table F4 for means and standard deviations.

The effects of scale labelling were directionally opposite to those found in the overall score ANCOVA, though the effect size was very small; participants using the linear scale labels performed better than those using the geometric scale labels (M(s.d.): 4.81 (1.58) versus 4.67 (1.59) respectively). This was confirmed by a Mann-Whitney *U* test (*p*=0.02).

The effects for format and format×key were the same directionally as the overall performance score ANCOVA effects: Text performed worst, standard matrix performed intermediately, and logarithmic matrix performed best; integrated information increased overall scores for the standard and logarithmic matrices, but decreased overall scores for the text.

These results should, however, be interpreted with caution, given the ceiling effect on the basic knowledge questions and violations of ANCOVA assumptions in these analyses.

**E2. Matrix Preference**

Three questions separately asked for participants’ preference between the format options, the scale labelling options, and the key options.

For format preference, among all participants, 42% preferred the standard matrix, 30% the logarithmic matrix, 13% the text, and 15% no preference (Supplementary Table F5). A chi-square test of independence demonstrated a significant relationship between format seen during tasks and format selected, *X^2^*(6, *N*=1273)=168.01, *p<*0.0001. Participants in the standard matrix condition were most likely to prefer the standard matrix (preferred by 59%, 95% CI=54%–63%). They were also more likely to prefer the logarithmic matrix (est.=22%, 95% CI=17%–27%) than the text (est.=7%, 95% CI=2%–12%). Participants in the logarithmic matrix condition were most likely to prefer the logarithmic matrix (est.=45%, 95% CI=41%–50%), followed by the standard matrix (est.=33%, 95% CI=29%–39%). Participants in the text condition were most likely to prefer the standard matrix (est.=35%, 95% CI=30%–40%).

For scale labelling preference, among all participants, 42% preferred the linear scale labels, 35% preferred the geometric scale labels, and 22% had no preference (Supplementary Table F6). A chi-square test of independence revealed a significant relationship between scale labelling seen during tasks and scale labelling selected, *X^2^(*2, *N*=1273)=9.68, *p=*0.008. Participants in the linear scale labelling condition were most likely to prefer the linear scale labelling (est.=45%, 95% CI=41%–49%). For participants in the geometric scale labelling condition, confidence intervals of the proportion preferring the linear scale labelling (est.=40%, 95% CI=35%–44%) and the proportion preferring the geometric scale labelling (est.=40%, 95% CI=35%–44%) overlapped.

For key preference, among all participants, 42% preferred the legend, 37% preferred the integrated information, and 15% had no preference (Supplementary Table F7). A chi-square test of independence again revealed a significant relationship between scale labelling seen during tasks and key selected, *X^2^(*2, *N*=1273)=29.33, *p<*.0001. Participants in the legend condition were most likely to prefer the legend (est.=57%, 95% CI=53%–61%), followed by the integrated information (est.=29%, 95% CI=25%–33%). Participants in the integrated condition, seemed as likely to prefer the legend (est.=43%, 95% CI=39%–47%) as they were to prefer the integrated information (est.=42%, 95% CI=38%–47%).

**E3. Subjective Feedback**

Three-way ANCOVAs, controlling for numeracy, were used to examine pre- and post-task subjective ratings on comprehension, ease of use and the differences between pre- and post-task scores on each, as well as confidence in using the format and explaining it to others.

For histograms of all subjective ratings, please see Supplementary Figs. G5-G16.

*E3.1. Self-reported comprehension ratings*

Both pre- and post-task self-reported comprehension ratings showed a main effect of format. (M(s.d.) for text, logarithmic matrix, and standard matrix: pre-task 4.40(1.80) versus 4.88(1.64) versus 5.09(1.64) respectively, F_2,1260_=19.45, *p*<0.0001, *f*=0.18 (90% CI=0.13–0.22); post-task 4.21(1.86) versus 4.88(1.54) versus 5.02(1.55) respectively, F_2,1260_=29.65, *p*<0.0001, *f*=0.22 (90% CI=0.17 – 0.26)). A Tukey’s posthoc revealed that the standard and logarithmic matrices were rated significantly higher than text (pre-task est.=0.64 (95% CI=0.42–0.86), *p*<0.0001 and est.=0.043 (95% CI=0.21–0.65), *p*=0.0004 respectively; post-task est.=0.77 (95% CI=0.55–0.99), *p*<0.0001 and est.=0.62 (95% CI=0.40–0.84), *p*<0.0001), but there was no significant difference between the standard and logarithmic matrices (pre-task *p*=0.13; post-task *p*=0.37)

There was also a main effect of key, with the legend rated higher than the integrated information (M(s.d.) pre-task 4.93 (1.64) versus 4.67 (1.78) respectively, F_1,1260_=8.53, *p*=0.004, *f*=0.08, (90% CI=0.04–0.13); post-task 4.85 (1.60) versus 4.57 (1.75) respectively, F_2,1260_=10.88, *p*=0.001, *f*=0.09 (90% CI=0.05–0.14)). There was no main effect of scale labelling (pre-task: F_1,1260_=1.46, *p*=0.227; post-task F_1,1260_=0.97 *p*=0.323). There was an interaction effect of format×key (pre-task F_2,1260_=8.42, *p*=0.0002, *f*=0.12 (90% CI=0.06–0.16); post-task F_2,1260_=8.45, *p*=0.0002, *f*=0.12 (90% CI=0.06–0.16)); descriptively, integrated information increased ratings for the standard matrix, but decreased ratings for both text and the logarithmic matrix.

*E3.2. ‘Ease of use’ ratings*

Both pre- and post-task ease of use ratings showed a main effect of format. M(s.d.) for text, logarithmic matrix, and standard matrix: pre-task 4.67 (1.74) versus 5.09 (1.50) versus 5.40 (1.44) respectively, F_2,1260_=22.99, *p*<0.0001, *f*=0.19, (90% CI=0.14–0.24); post-task 4.65 (1.72) versus 5.19 (1.52) versus 5.31 (1.49) respectively, F_2,1260_=20.49, *p*<0.0001, *f*=0.18 (90% CI=0.13–0.23). A Tukey’s posthoc revealed that the logarithmic and standard matrices were rated significantly higher than text (pre-task est.=0.39 (95% CI=0.17–0.61), *p*<0.001 and est.=0.70 (95% CI=0.48–0.92), *p*<0.001 respectively; post task est.=0.51 (95% CI=0.29–0.73), *p*<0.0001 and est.=0.63 (95% CI=0.41–0.85), *p*<0.0001 respectively). The standard matrix was rated significantly higher than the logarithmic matrix pre-task (est.=0.31 (95% CI=0.09–0.53), *p*=0.009), but not post-task (*p*=0.48).

There was also a main effect of key, with the legend condition rated higher than the integrated condition (M(s.d.) pre-task: 5.21 (1.53) versus 4.92 (1.63) respectively; post-task: 5.19 (1.55) versus 4.93 (1.63) respectively; pre-task F_1,1260_=12.05, *p*=0.001; effect size *f*=0.10, 90% CI: 0.05 – 0.14; post-task F_1,1260_=0, *p*=0.002; effect size *f*=0.09, 90% CI: 0.04 – 0.13). There was no main effect of scale labelling (pre-task: F_1,1260_=0.08, *p*=0.781; post-task: F_1,1260_<0.01, *p*=0.948).

There was additionally an interaction effect of format×key for pre-task scores (F_2,1260_=8.94, *p*=0.0001; effect size *f*=0.12, 90% CI: 0.07 – 0.16) and post-task scores (F_2,1260_=7.68, *p*=0.0004; effect size *f*=0.11, 90% CI: 0.06 – 0.15). Descriptively, for pre-task scores, integrated information increased ratings for the standard matrix, but decreased ratings for both text and the logarithmic matrix. For post-task scores the integrated condition was rated lower than the legend condition for all formats, but the difference was much larger for text.

*E3.3. Confidence using and explaining the format*

Scores on both post-task ‘confidence using format oneself’ and ‘confidence explaining format to others’ showed a main effect of format. M(s.d.) for text, logarithmic matrix, and standard matrix: self 4.08 (1.87) versus 4.65 (1.67) versus 4.76 (1.68) respectively, F_2,1260_=18.51, *p*<0.0001, *f*=0.17 (90% CI=0.12–0.22); others 3.92 (1.88) versus 4.70 (1.68) versus 4.81 (1.70) respectively, F_2,1260_=32.61, *p*<0.0001, *f*=0.23 (90% CI=0.18–0.27). A Tukey’s posthoc revealed that both the standard and logarithmic matrices were rated significantly higher than the text format (self est.=0.66 (95% CI=0.42–0.90), *p*<0.0001 and est.=0.55 (95% CI=0.31–0.79), *p*<0.0001) respectively; other est.=0.87 (95% CI=0.63–1.11), *p*<0.0001 and est.=0.76 (95% CI=0.52–1.00), *p*<0.0001 respectively). There were no significant differences between the standard and logarithmic matrices (self *p*=0.60; other *p*=0.61).

There was also a main effect of key, with legend condition rated higher than integrated (M(s.d.) self: 4.66 (1.69) versus 4.35 (1.81) respectively; others: 4.62 (1.75) versus 4.36 (1.83) respectively; self: F_1,1260_=11.19, *p*=0.001, effect size *f*=0.09, 90% CI: 0.05 – 0.14; others: F_1,1260_=8.07, *p*=0.005; effect size *f*=0.08, 90% CI: 0.03 – 0.13). There was no main effect of scale labelling (self: F_1,1260_=0.02, *p*=0.902; others: F_1,1260_=0.05, *p*=0.823).

There was additionally an interaction effect of format×key for ‘self’ ratings (F_2,1260_=3.66, *p*=0.026; effect size *f*=0.08, 90% CI: 0.02 – 0.12) and for ‘other’ ratings (F_2,1260_=5.88, *p*=0.003; effect size *f*=0.10, 90% CI: 0.04 – 0.14). Descriptively, for ‘self’ ratings, the integrated condition was rated lower than legend for all formats, but the difference was much larger for text. For ‘other’ ratings, integrated information increased ratings for the standard matrix, but decreased ratings for both text and the logarithmic matrix.

*E3.4. Participants with Prior Risk Matrix Experience*

The 369 participants who reported some experience with using risk matrices (“yes, rarely”, “yes, sometimes”, and “yes, often”) were analysed as a subset. Basic knowledge and risk comparison scores of these participants in each condition are reported in Table S8 and Table S9.

A three-way ANCOVA on risk comparison score, controlling for numeracy revealed a main effect of format. M(s.d.) for logarithmic matrix, text, and standard matrix: 8.33 (3.56) versus 9.02 (3.87) versus 9.32 (4.40) respectively; F_2,369_=3.15, *p*=0.044, *f*=0.13 (90% CI=0.02–0.21)). A Tukey’s posthoc revealed no significant differences between any formats.

There was also a main effect of scale labelling, with participants shown the geometric scale labelling performing better than those shown the linear scale labelling, (M(s.d.): 9.92 (4.19) versus 7.88 (3.46) respectively; F_1,369_=34.58, *p*<0.0001, *f*=0.31 (90% CI=0.22–0.40)). There was no main effect of key (F_1,369_=1.00, *p*=0.317), and no interaction effects.

The same test on overall performance score, controlling for numeracy, revealed a main effect of scale labelling, with the geometric scale labelling performing better than the linear scale labelling, (M(s.d.): 14.22 (5.43) versus 12.67 (4.32) respectively; F_1,369_=14.52, *p*=0.0002, *f*=0.20 (90% CI=0.11–0.29)). There was no main effect of format (F_2,369_=2.19, *p*=0.114), or of key (F_1,369_=0.42, *p*=0.515), and there were no interaction effects.

The same test on basic knowledge score, controlling for numeracy, revealed a main effect of format. M(s.d.) for text, standard matrix, and logarithmic matrix: 4.19 (1.78) versus 4.69 (1.66) versus 4.73 (1.55) respectively; F_1,369_=6.23, *p*=0.002, *f*=0.19 (90% CI=0.09–0.27). A Tukey’s posthoc revealed that participants shown the standard and logarithmic matrices performed significantly better than those shown the text format (est.=0.43 (95% CI=0.10–0.76), *p*=0.03 versus est.=0.47 (95% CI=0.13–0.80), *p*=0.01 respectively)

There was also a main effect of scale labelling, with participants shown the linear scale labelling performing better than those shown the geometric scale labelling (M(s.d): 4.79 (1.60) versus 4.31 (1.71) respectively; F_1,369_=12.31, *p*<0.001, *f*=0.19 (90% CI=0.10–0.27)). There was no main effect of key (F_1,369_=0.35, *p*=0.55), and there were no interaction effects.

The results of these ANCOVAs, however, should be interpreted with caution, as taking a subset of participants inevitably reduces the statistical power of the tests. Particular caution should be used for the basic knowledge score results, as the ceiling effect produced non-normal data.

**E4. Interactions with Numeracy**

Exploratory repeats of the analyses in section 3.2.1 of the main manuscript, but in which format×numeracy interaction terms were included in the models, did not yield significant interactions between format and numeracy.

**E5. Likelihood vs. Impact**

As in Experiment 1, we found that the tendency to select likelihood/impact reduction remained consistent across all conditions. A one-sample t-test, with a reference value of 0, revealed a mean prioritization score of 0.08 (95% CI: 0.05 – 0.11), indicating an overall tendency towards reducing impact, *t*(1272)=5.55, *p*<0.0001. See Supplementary Fig. G4. A three-way ANCOVA showed no main effect of format (F_2,1260_=0.35, *p*=0.704), scale labelling (F_1,1260_=0.31, *p*=0.573), or key (F_1,1260_=1.50, *p*=0.221). There were no significant interaction effects, and numeracy was not a significant covariate.

# **APPENDIX F. Supplementary Tables**

**Table S1.** Demographic Information of Participants for Experiments 1 and 2

| **Characteristics of Participants** | **Experiment 1**  *total n*=1426  n (rounded %) | **Experiment 2**  *total n*=1273  n (rounded %) |
| --- | --- | --- |
| **Sex:**  Male  Female | 685 (48.3%)  737 (51.7%) | 611 (48.3%)  658 (51.7%) |
| **Age:**  18-24  25-34  35-44  45-54  55-64  65+ | 174 (12.2%)  263 (18.4%)  273 (19.1%)  271 (19.0%)  240 (16.8%)  205 (14.4%) | 155 (12.2%)  236 (18.5%)  244 (19.2%)  242 (19.0%)  213 (16.7%)  283 (14.4%) |
| **Education level:**  None  GCSE  A-Level  Higher Ed.  Bachelors  Master’s  PhD  No Answer | 57 (4%)  303 (21.2%)  293 (20.5%)  149 (10.4%)  404 (28.3%)  180 (12.6%)  39 (2.7%)  1 (0.1%) | 60 (4.7%)  236 (18.5%)  265 (20.8%)  142 (11.2%  406 (31.9%)  140 (11.0%)  21 (1.7%)  3 (0.2%) |
| **Numeracy:**  1  2  3  4  5  6  7  8 | 65 (4.6%)  110 (7.7%)  188 (13.2%)  274 (19.2%)  269 (18.9%)  155 (17.9%)  97 (6.8%)  168 (11.8%) | 58 (4.6%)  124 (9.7%)  168 (13.2%)  236 (18.5%)  239 (18.8%)  202 (15.9%)  91 (7.2%)  115 (12.2%) |
| **Previous experience with risk matrices:**  No, never  Yes, rarely  Yes, sometimes  Yes, often | 1022 (71.7%)  212 (14.9%)  154 (10.8%)  38 (2.7%) | 904 (71.0%)  182 (14.3%)  155 (12.2%)  32 (2.5%) |

**Supplementary Table F2.** Experiment 1; Means (Standard Deviations) of Basic Knowledge Scores (out of 8) and of Risk Comparison Scores (out of 18) by Condition, for Participants with Prior Risk Matrix Experience

|  |  | **Format** | | | |
| --- | --- | --- | --- | --- | --- |
|  | Standard Matrix  (*n*=100) | | Square Matrix (*n*=99) | Logarithmic Matrix (*n=*100) | Text  (*n*=105) |
| Basic Knowledge Score /8 | 6.69 (2.09) | | 6.65 (1.19) | 6.82 (1.97) | 6.84 (1.88) |
| Comparison Score / 18 | 8.32 (3.17) | | 7.34 (3.08) | 8.18 (3.05) | 7.91 (3.72) |

**Supplementary Table F3.** Experiment 1; Cross Table of Format Seen by Format Preferred, Showing Number of Participants

|  |  | **Format Seen** | | | | | |  |
| --- | --- | --- | --- | --- | --- | --- | --- | --- |
| **Format Preferred** | Standard Matrix  (*n*=341) | | Square Matrix  (*n*=364) | | Logarithmic Matrix  (*n*=362) | | Text (*n*=359) | Total |
| Standard Matrix | 126 | | 51 | 48 | | 66 | | 291 |
| Square Matrix | 94 | | 170 | 127 | | 79 | | 470 |
| Logarithmic Matrix | 56 | | 59 | 118 | | 68 | | 301 |
| Text | 32 | | 46 | 33 | | 101 | | 212 |
| None of the Above | 33 | | 38 | 36 | | 45 | | 152 |

**Supplementary Table F4.** Experiment 2; Means (Standard Deviations) of Basic Knowledge Scores (out of 6) by Condition

|  |  | **Format** | | |
| --- | --- | --- | --- | --- |
| **Scale** | **Key** | Standard Matrix | Logarithmic Matrix | Text |
| 1-5 | Legend | 4.88 (1.53)  *n*=98 | 5.07 (1.42)  *n*=100 | 4.41 (1.73)  *n* =111 |
|  | Integrated | 4.93 (1.62)  *n*=119 | 5.15 (1.25)  *n* =117 | 4.38 (1.80)  *n*=93 |
| 1-625 | Legend | 4.79 (1.56)  *n=*107 | 4.82 (1.62)  *n*=116 | 4.59 (1.61)  *n*=95 |
|  | Integrated | 4.89 (1.48)  *n*=98 | 4.91 (1.35)  *n*=113 | 3.90 (1.72)  *n*=106 |

**Supplementary Table F5.** Experiment 2; Cross Table Format Seen by Format Preferred, by Number of Participants

|  | **Format Seen** | | |
| --- | --- | --- | --- |
| **Format Preferred** | Standard Matrix  (*n*=422) | Logarithmic Matrix  (*n*=446) | Text (*n*=405) |
| Standard Matrix  (*n*=538) | 247 | 149 | 142 |
| Logarithmic Matrix  (*n*=378) | 91 | 202 | 85 |
| Text  (*n=*161) | 28 | 34 | 99 |
| None of the Above  (*n*=196) | 56 | 61 | 79 |

**Supplementary Table F6.** Experiment 2; Cross Table Scale Seen by Scale Preferred, by Number of Participants

|  | **Scale Seen** | | |
| --- | --- | --- | --- |
| **Scale Preferred** | 1-5 Scale  (*n*=638) | 1-625 Scale  (*n*=635) |  |
| 1-5 Scale  (*n*=538) | 287 | 251 |  |
| 1-625 Scale  (*n=450*) | 199 | 251 |  |
| None of the Above  (*n*=285) | 152 | 133 |  |

**Supplementary Table F7.** Experiment 2; Cross Table Key Seen by Key Preferred, by Number of Participants

|  | **Key Seen** | | |
| --- | --- | --- | --- |
| **Key Preferred** | Legend  (*n*=627) | Integrated  (*n*=596) |  |
| Legend  (*n*=587) | 360 | 227 |  |
| Integrated  (*n=*457) | 183 | 274 |  |
| None of the Above  (*n*=179) | 84 | 95 |  |

**Supplementary Table F8.** Experiment 2; Means (Standard Deviations) of Basic Knowledge Scores (out of 6) by Condition, for Participants with Prior Risk Matrix Experience

|  |  | **Format** | | |
| --- | --- | --- | --- | --- |
| **Scale** | **Key** | Standard Matrix | Logarithmic Matrix | Text |
| Linear | Legend | 5.07 (1.38)  *n*=27 | 5.15 (1.39)  *n*=41 | 4.30 (1.84)  *n*=27 |
|  | Integrated | 4.61 (1.78)  *n*=36 | 4.91 (1.82)  *n*=35 | 4.54 (1.77)  *n*=28 |
| Geometric | Legend | 4.42 (1.70)  *n=*33 | 4.19 (1.82)  *n*=32 | 4.32 (1.65)  *n*=25 |
|  | Integrated | 4.77 (1.70)  *n*=26 | 4.53 (1.46)  *n*=30 | 3.66 (1.82)  *n*=29 |

**Supplementary Table F9.** Experiment 2; Means (Standard Deviations) of Risk Comparison Scores (out of 18) by Condition, for Participants with Prior Risk Matrix Experience

|  |  | **Format** | | |
| --- | --- | --- | --- | --- |
| **Scale** | **Key** | Standard Matrix | Logarithmic Matrix | Text |
| Linear | Legend | 8.00 (3.16)  *n*=27 | 7.49 (3.06)  *n*=41 | 7.33 (4.01)  *n*=27 |
|  | Integrated | 7.82 (3.71)  *n*=36 | 7.63 (3.21)  *n*=35 | 9.25 (3.67)  *n*=28 |
| Geometric | Legend | 10.19 (5.21)  *n=*33 | 9.00 (3.93)  *n*=32 | 10.48 (3.85)  *n*=25 |
|  | Integrated | 11.38 (4.14)  *n*=26 | 9.60 (3.83)  *n*=30 | 9.10 (3.52)  *n*=29 |

# **APPENDIX G. Supplementary Figures**

**Supplementary Fig. G1:** Comprehension questions. Grids shown here only indicate the positions of risks A and B on the matrices; see Figs. 1-4 (main manuscript) for examples of actual stimuli with labelled axes.

| *Question* | *Positions of risks A and B (not stimuli; see figure caption)* | *Response choices* |
| --- | --- | --- |
| “Consider risk A, shown above. What is the likelihood of risk A?”  “Consider risk A, as shown above. What is the potential impact of risk A?”  [Both questions asked on same page for each stimulus] | 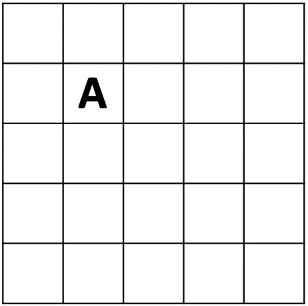 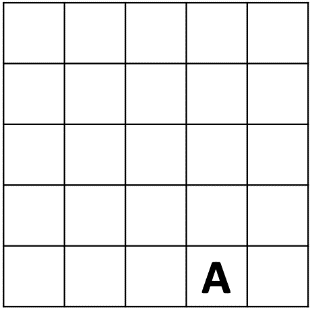 | 1 / 2 / 3 / 4 / 5  [For linear scale stimuli]  1 / 5 / 25 / 125 / 625  [For the geometric scale stimuli]  1 / 2 / 3 / 4 / 5  [For linear scale stimuli]  1 / 5 / 25 / 125 / 625  [For the geometric scale stimuli] |
| “Consider risk A, shown above. What is the likelihood of risk A?”  “Consider risk A, as shown above. What is the potential impact of risk A?”  [Both questions asked on same page for each stimulus] | 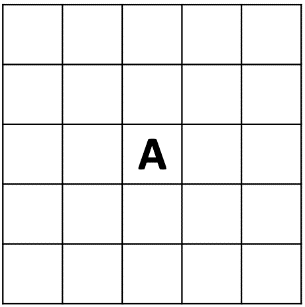 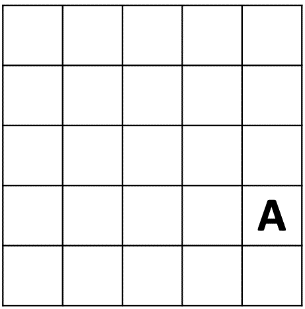 | Less than 0.2% / 0.2% to 1% / 1% to 5% / 5% to 25% / Over 25%  Limited / Minor / Moderate / Significant / Catastrophic |
| “Consider risks A and B, shown above. Which risk has the greater likelihood: risk A, or risk B?” (2 questions) | 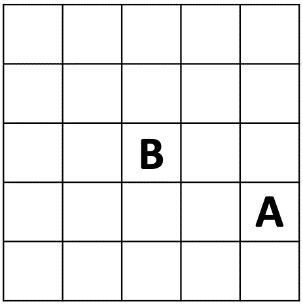 | Risk A / Risk B / Equal likelihood |
| “Consider risks A and B, shown above. Which risk has the greater impact: risk A, or risk B?” (2 questions) | 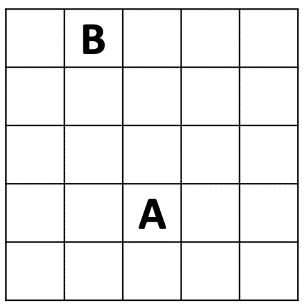 | Risk A / Risk B / Equal impact |
| “Which position on the grid above represents the greatest possible risk?” (For matrix stimuli; 1 question)  “Which position on the grid above represents the smallest possible risk?” (For matrix stimuli; 1 question)  [Questions removed from analysis for Exp2 due to experiment design/coding error.] | The same matrix format as previous questions shown, without any risks marked on it. | Top left / Bottom left / Top right / Bottom right / None of these |
| “Which combination of impact and likelihood represents the greatest possible risk?” (For text stimuli; 1 question)  “Which combination of impact and likelihood represents the smallest possible risk?” (For text stimuli; 1 question)  [Questions removed from analysis for Exp2 due to experiment design/coding error.] | The same text format as previous questions shown, with X and Y in the spaces where likelihood and impact numbers were ordinarily provided. | X=5, Y=1 / X=1, Y=1 / X=5, Y=5 / X=1, Y=5 / None of these |

**Supplementary Fig. G2.** Risk comparison questions. Grids shown here only indicate the positions of risks A and B on the matrices; see Figs. 1-4 (main manuscript) for examples of actual stimuli with labelled axes.

| *Question Text* | *Positions of risks A and B (not stimuli; see figure caption)* | *Response choices* |
| --- | --- | --- |
| “Consider risks A and B, shown above. How many times more likely is risk A than risk B?” (3 questions)  “Consider risks A and B, shown above. How many times more of an impact will risk A have than risk B?” (3 questions)  [Both questions asked on same page for all stimuli.] | 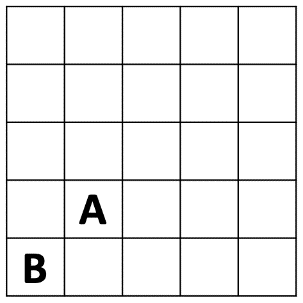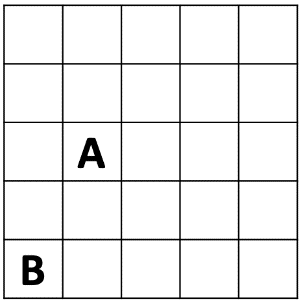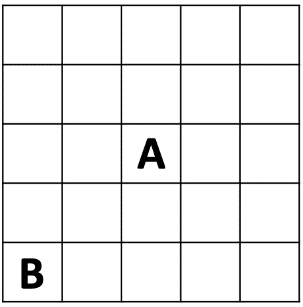 | Two times / Three times / Five times / Seven times / Ten times / Twenty-five times  Two times / Three times / Five times / Seven times / Ten times / Twenty-five times |
| “Local emergency planners have been working on a plan to reduce the likelihood of two flood risks, risk A and risk B. The likelihood of risk A and risk B before and after their work are shown above. Which risk has had its likelihood decreased the most?” (3 questions) | [‘Before’ on left; ‘after’ on right]  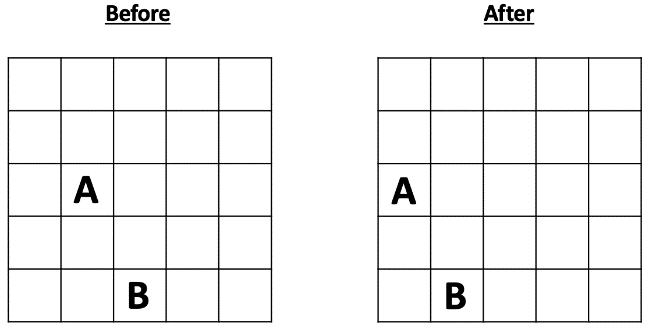  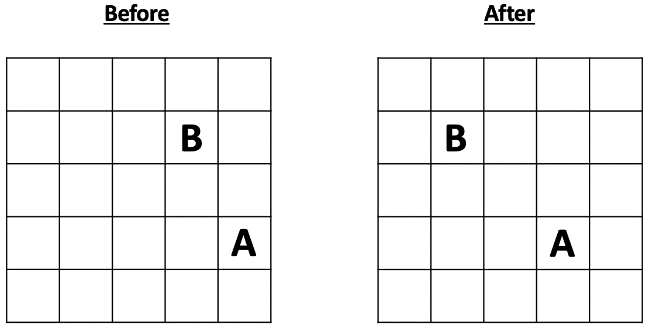  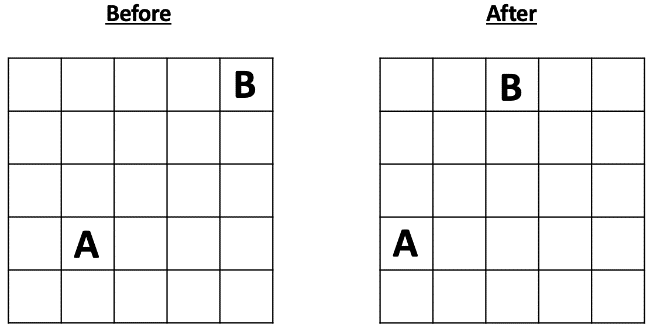 | Risk A / Risk B / Equal decrease |
| “Local emergency planners have been working on a plan to reduce the potential impact of two flood risks, risk A and risk B. The potential impact of risk A and risk B before and after their work are shown above. Which risk has had its potential impact decreased the most?” (3 questions) | [‘Before’ on left; ‘after’ on right]  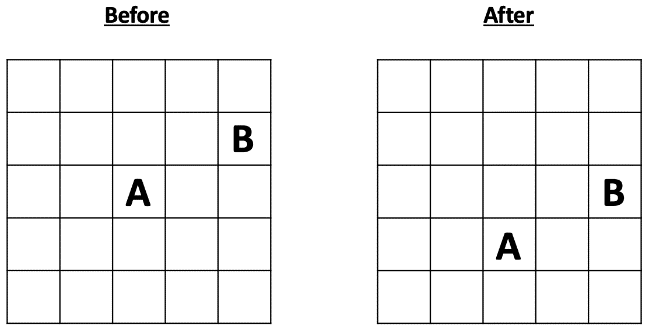  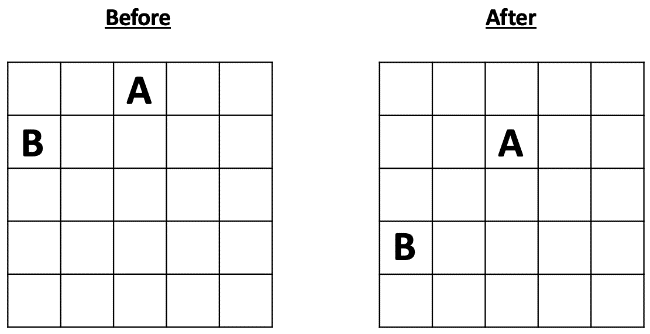­  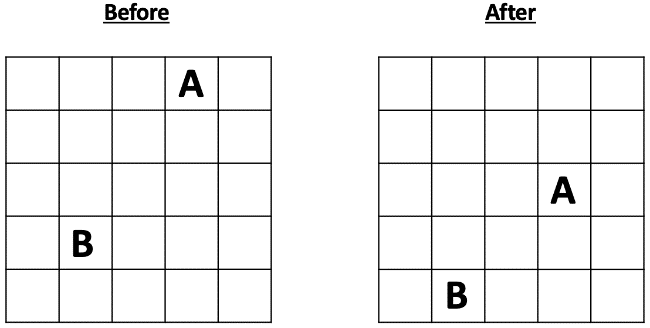 | Risk A / Risk B / Equal decrease |
| “Consider two flood risks, risk A and risk B, shown above. You are an emergency planner for the local government, and you have some money you can use to decrease the likelihood of either risk A or risk B, by the amount shown. Both risks cost the same to decrease, and you can only afford to decrease one. Do you decrease the likelihood of risk A or risk B?” (3 questions) | [‘Before’ on left; ‘after’ on right]  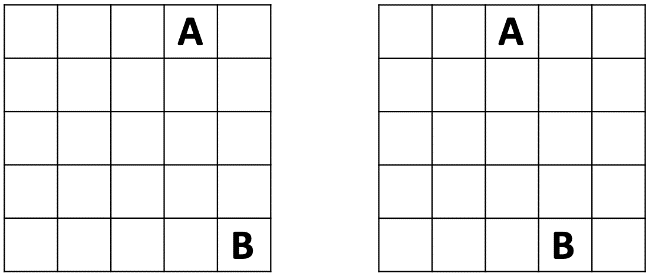  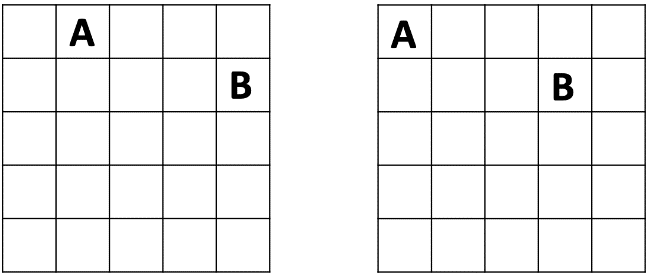  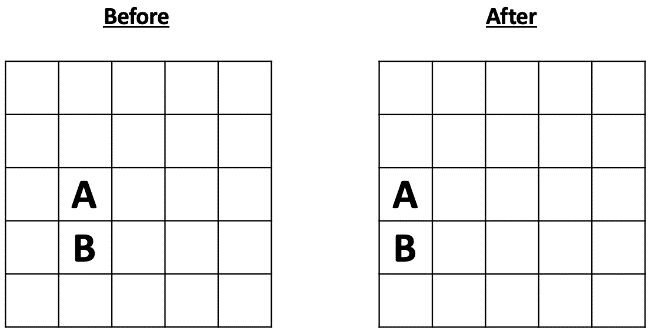 | Risk A / Risk B / Impossible to choose |
| “Consider two flood risks, risk A and risk B, shown above. You are an emergency planner for the local government, and you have some money you can use to decrease the potential impact of either risk A or risk B, by the amount shown. Both risks cost the same to decrease, and you can only afford to decrease one. Do you decrease the potential impact of risk A or risk B?” (3 questions) | [‘Before’ on left; ‘after’ on right]  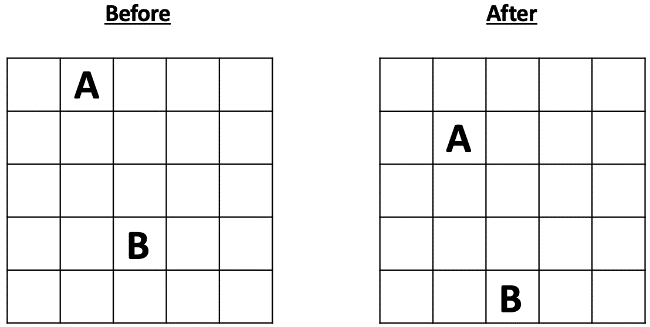  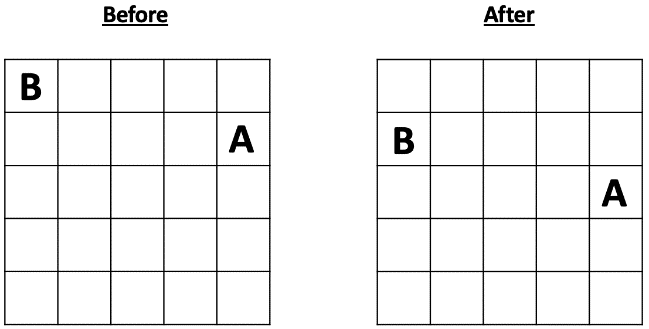  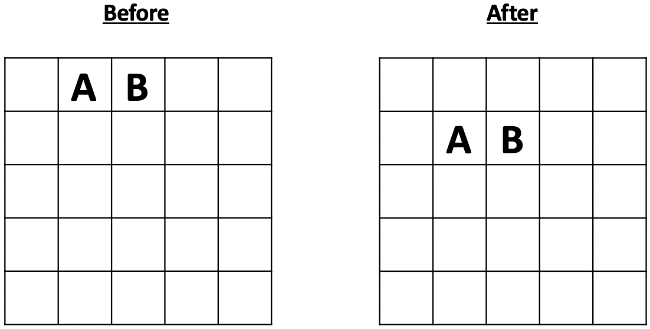 | Risk A / Risk B / Impossible to choose |

**Supplementary Fig. G3.** Relationship between numeracy and risk comparison scores for each format in Experiment 1.

**
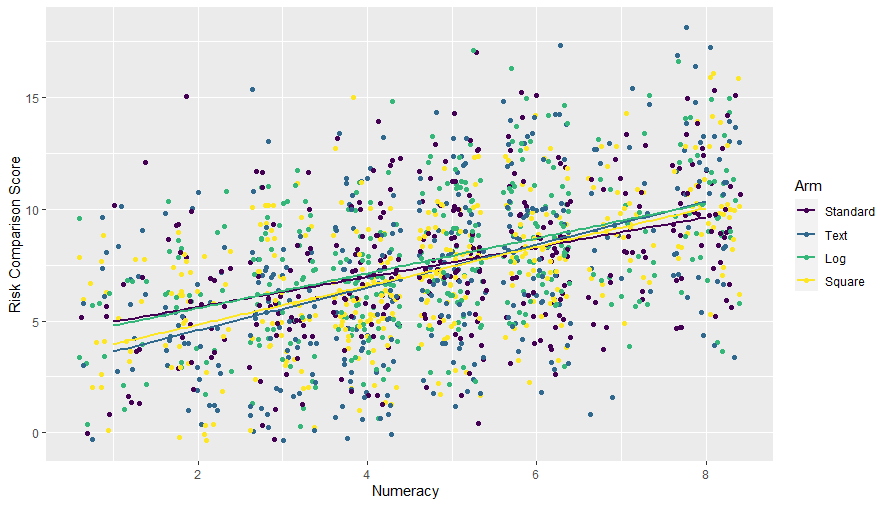
**

**Supplementary Fig. G4.** Graph showing distribution of all participants by prioritization score (1=maximum impact prioritization/participant always chose to reduce impact, -1=maximum likelihood prioritization/participant always chose to reduce likelihood) in Experiment 2.


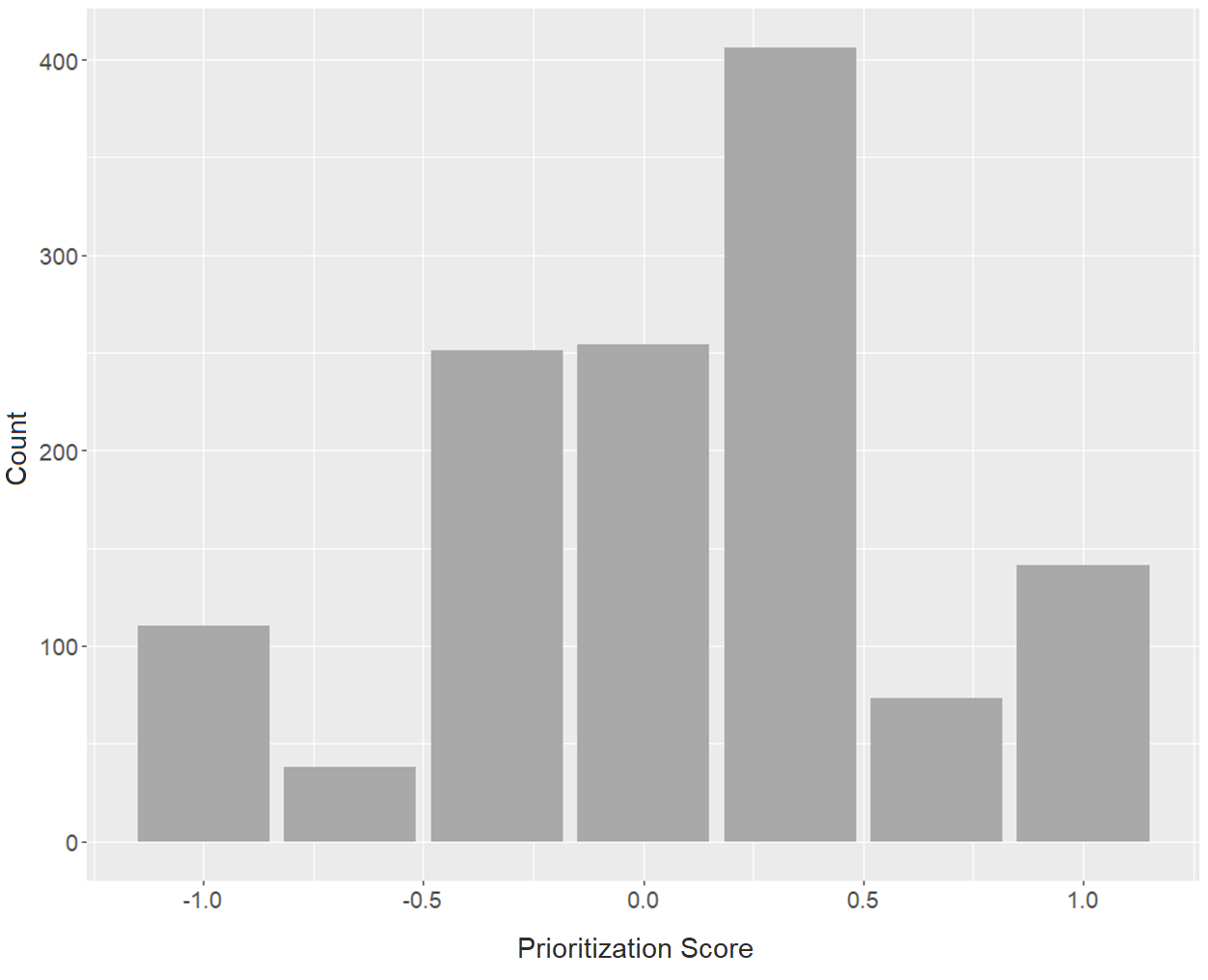


**Experiment 1**

**Supplementary Fig. G5.** Experiment 1; Distribution of Pre-Task and Post-Task Ease of Comprehension Ratings, by Format (means indicated by line)


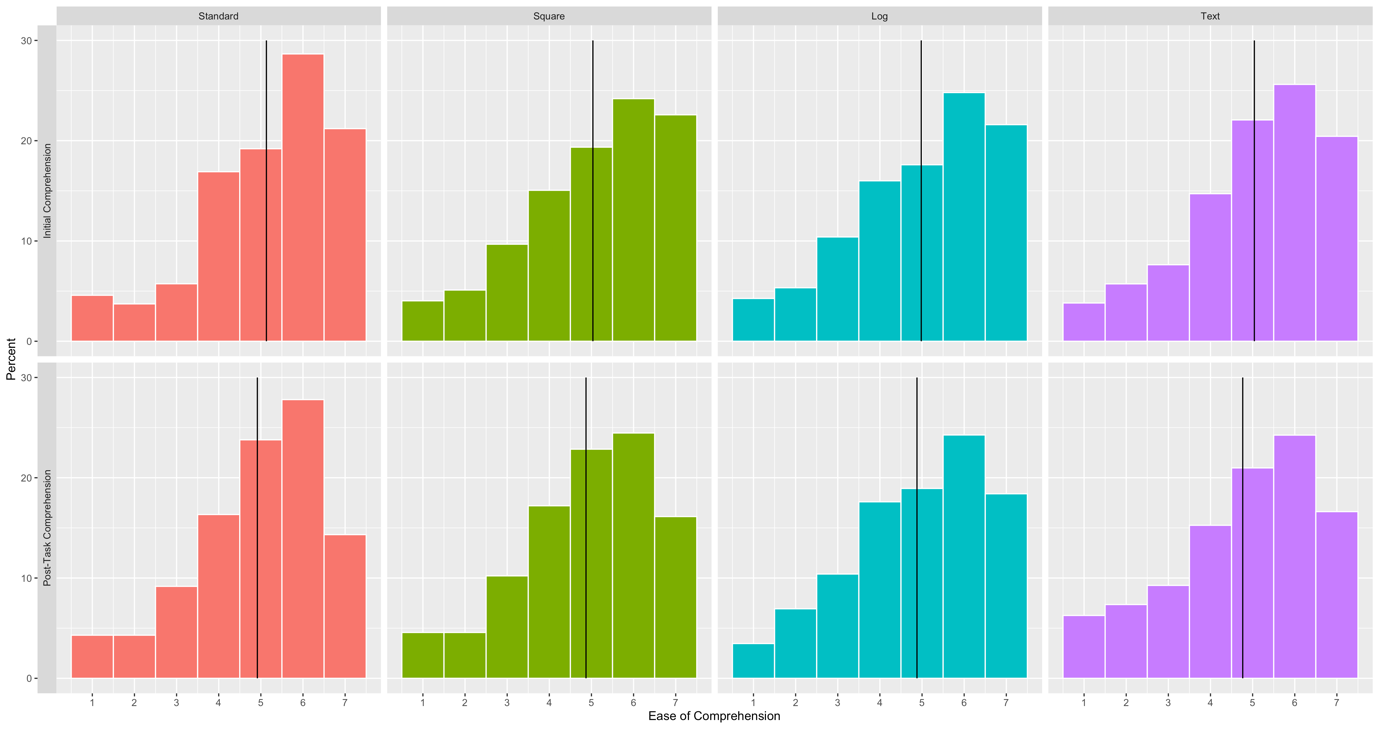


**Supplementary Fig. G6.** Experiment 1; Distribution of Pre-Task and Post-Task Ease of Use Ratings, by Format (means indicated by line)


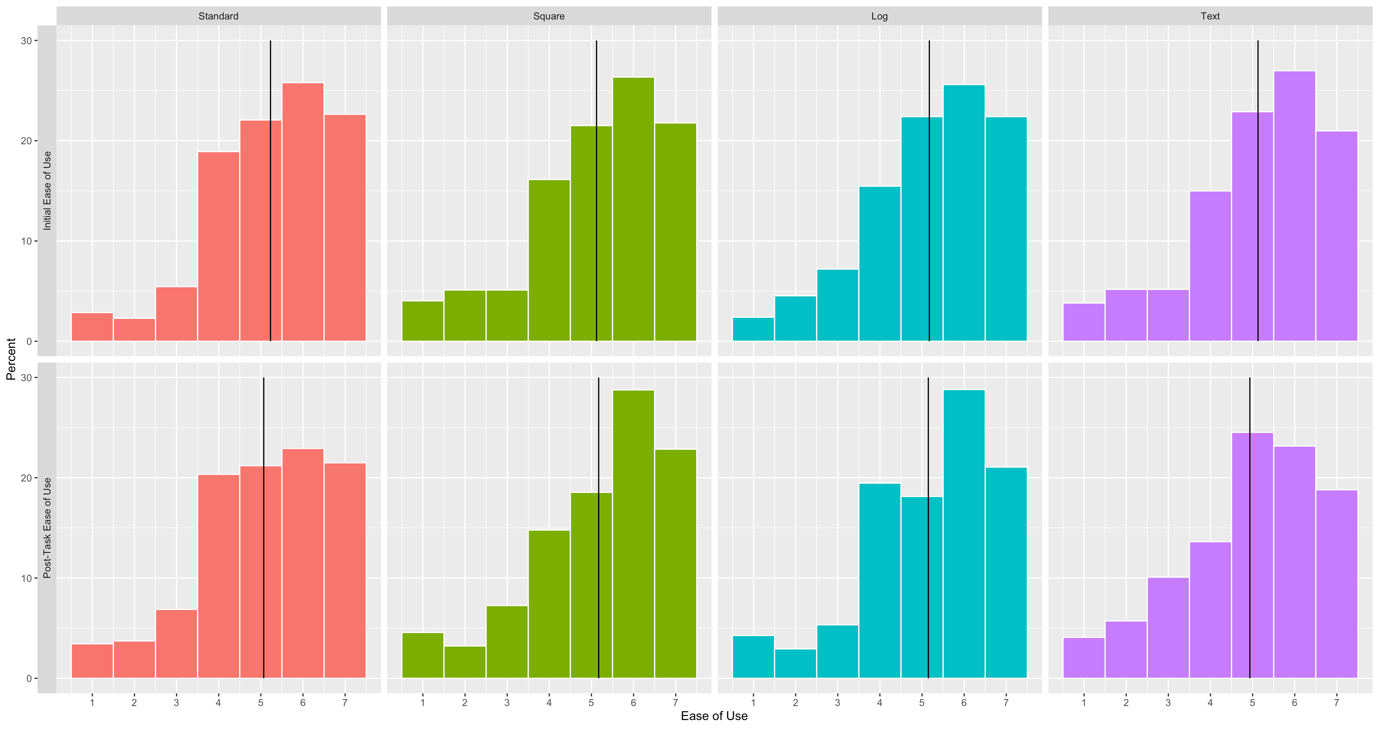


**Supplementary Fig. G7:** Experiment 1; Distribution of Post-Task Ease of ‘Confidence Using Format Oneself’ and ‘Confidence Explaining Format to Others’ Ratings, by Format (means indicated by line)


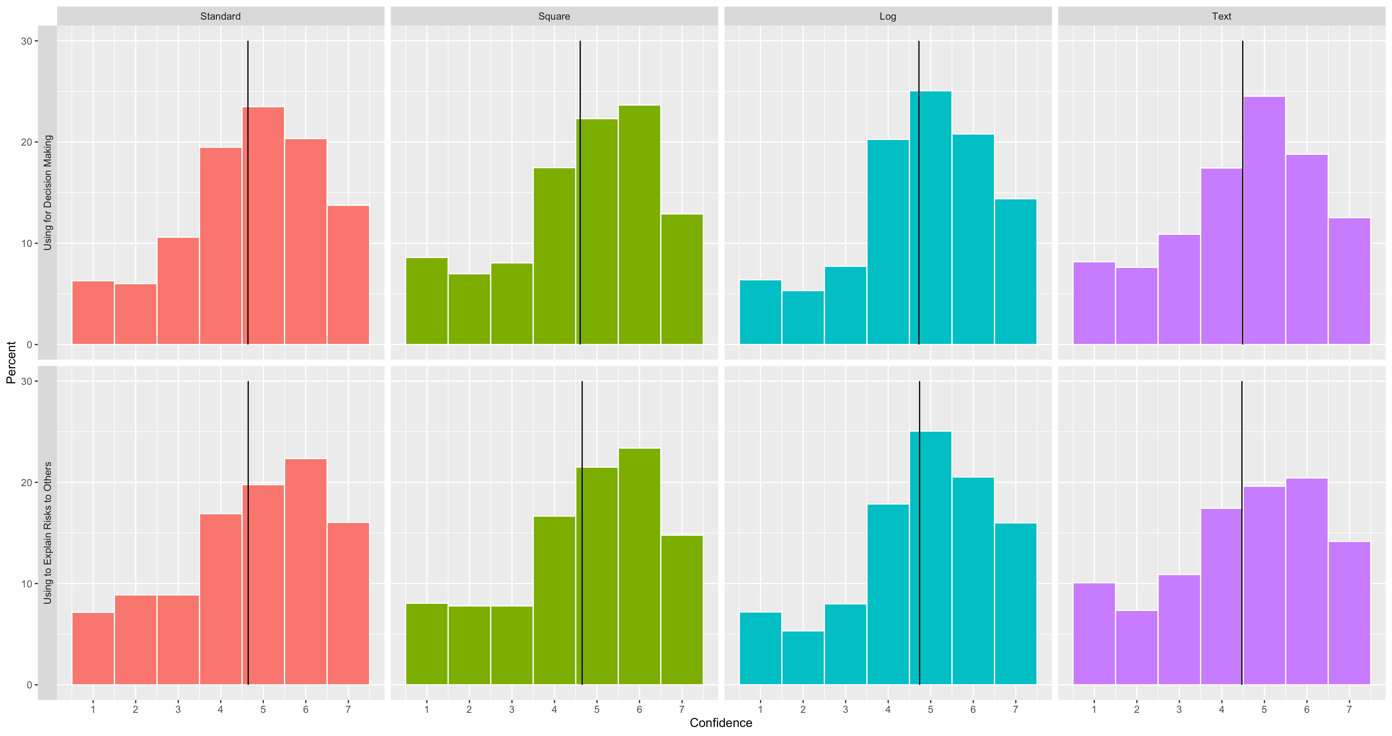


**Experiment 2**

**Supplementary Fig. G8.** Experiment 2; Distribution of Pre-Task and Post-Task Ease of Comprehension Ratings, by Format (means indicated by line)


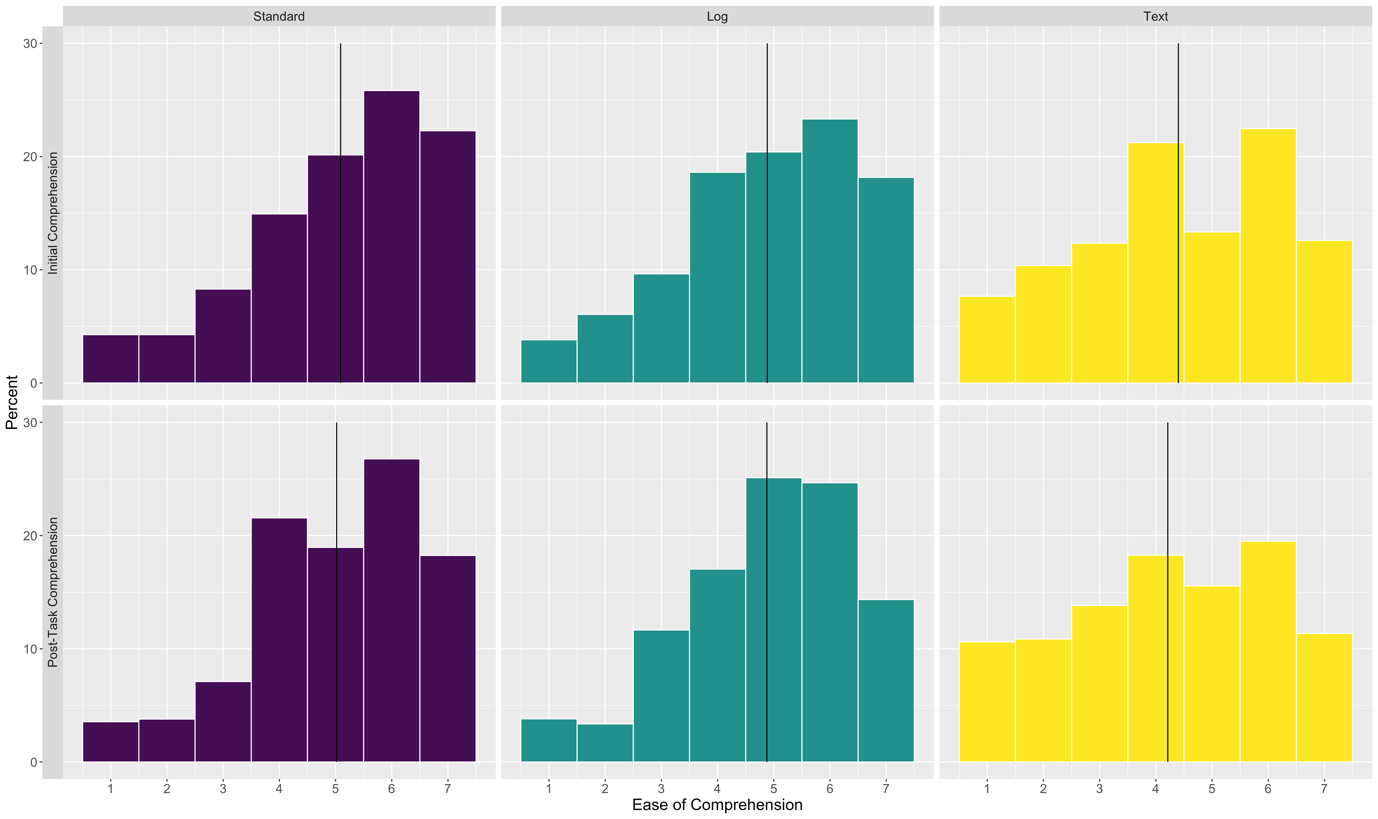


**Supplementary Fig. G9.** Experiment 2; Distribution of Pre-Task and Post-Task Ease of Comprehension Ratings, by Scale (means indicated by line)


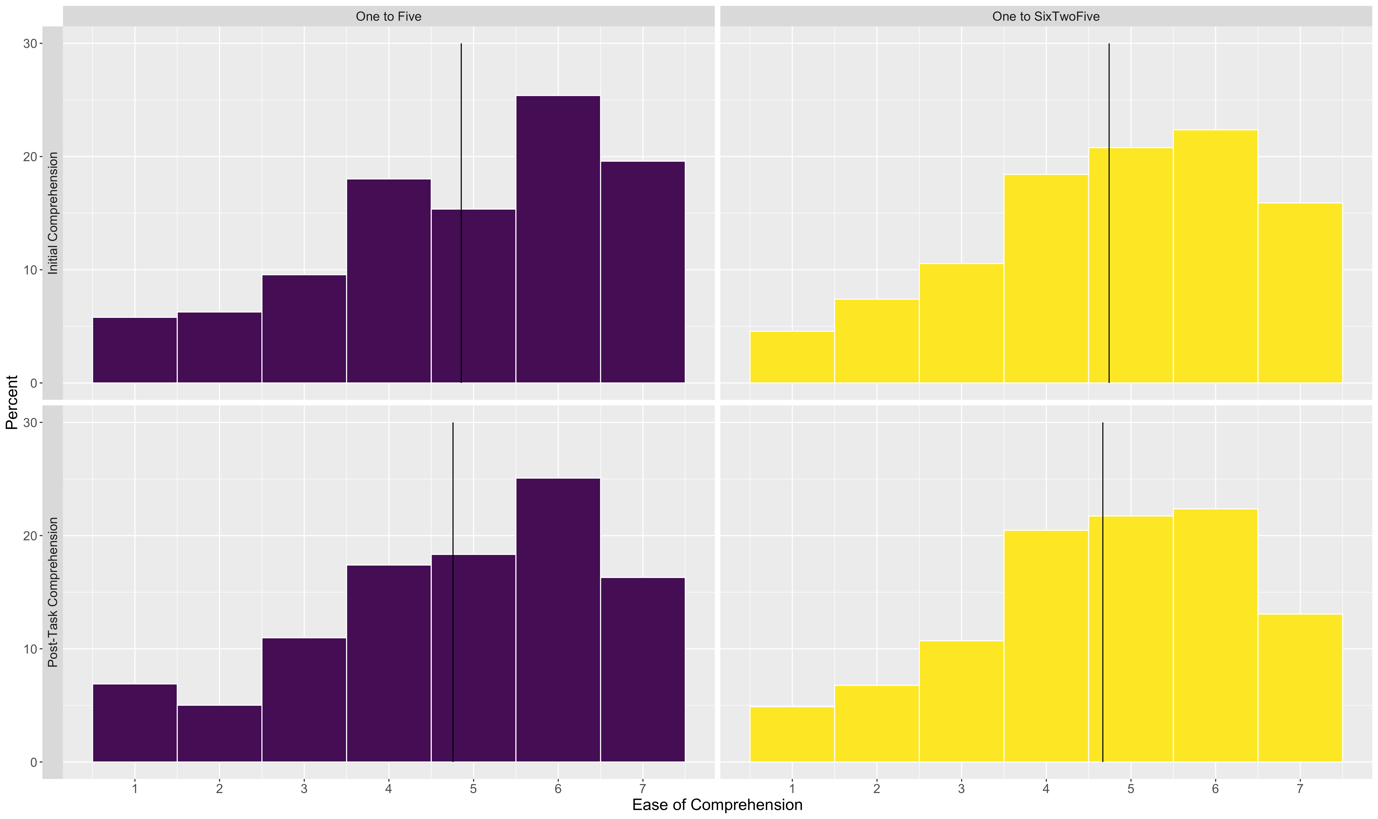


**Supplementary Fig. G10.** Experiment 2; Distribution of Pre-Task and Post-Task Ease of Comprehension Ratings, by Key (means indicated by line)


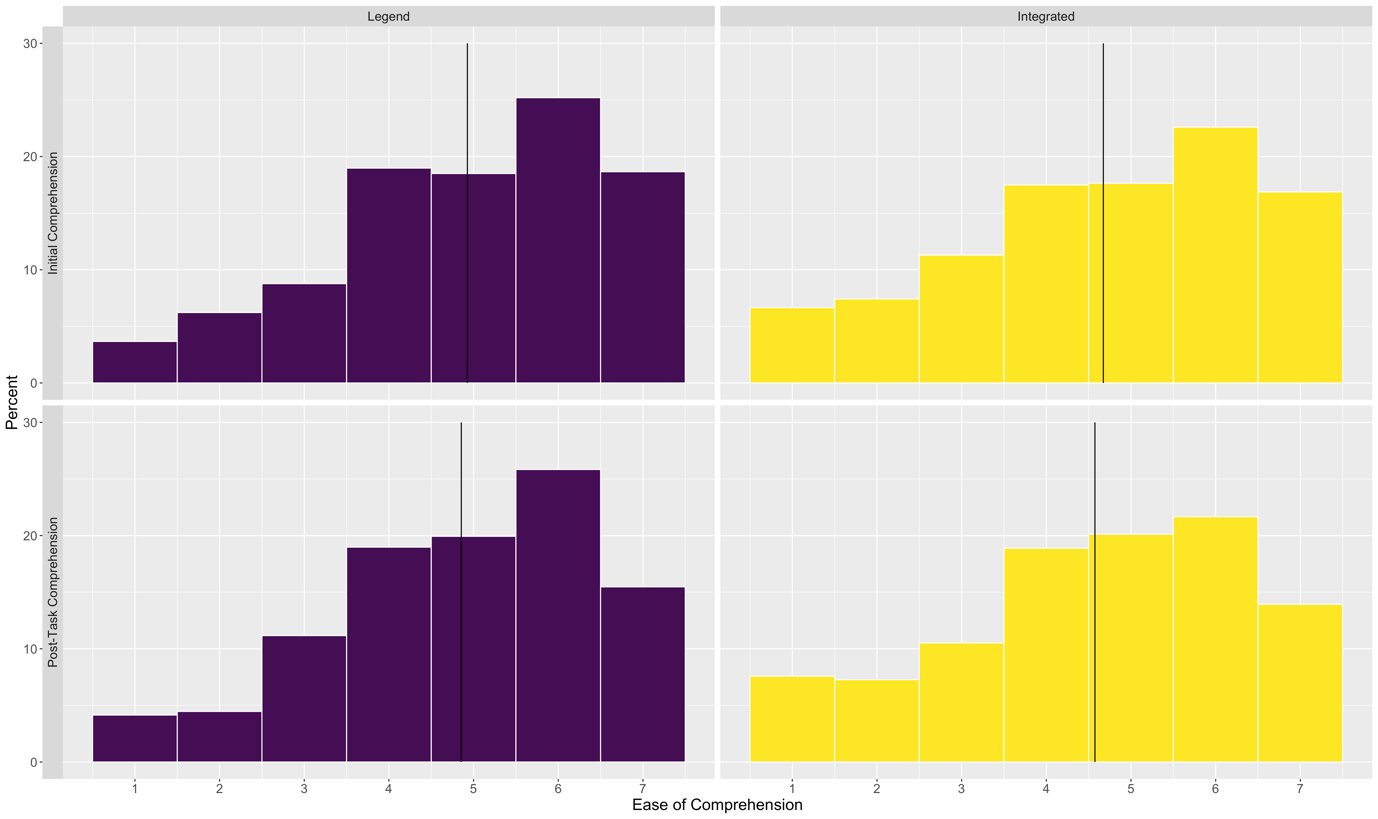


**Supplementary Fig. G11.** Experiment 2; Distribution of Pre-Task and Post-Task Ease of Use Ratings, by Format (means indicated by line)


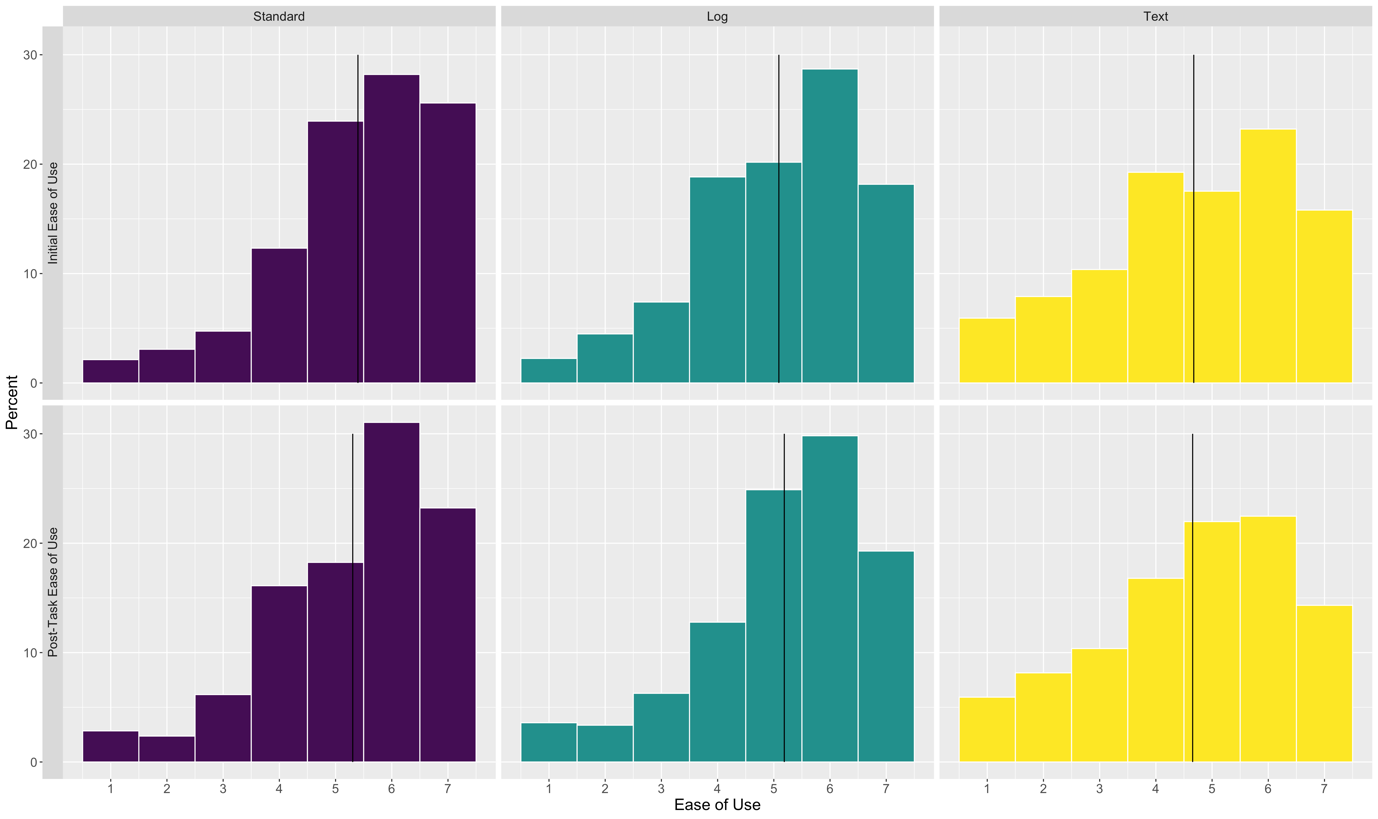


**Supplementary Fig. G12.** Experiment 2; Distribution of Pre-Task and Post-Task Ease of Use Ratings, by Scale (means indicated by line)


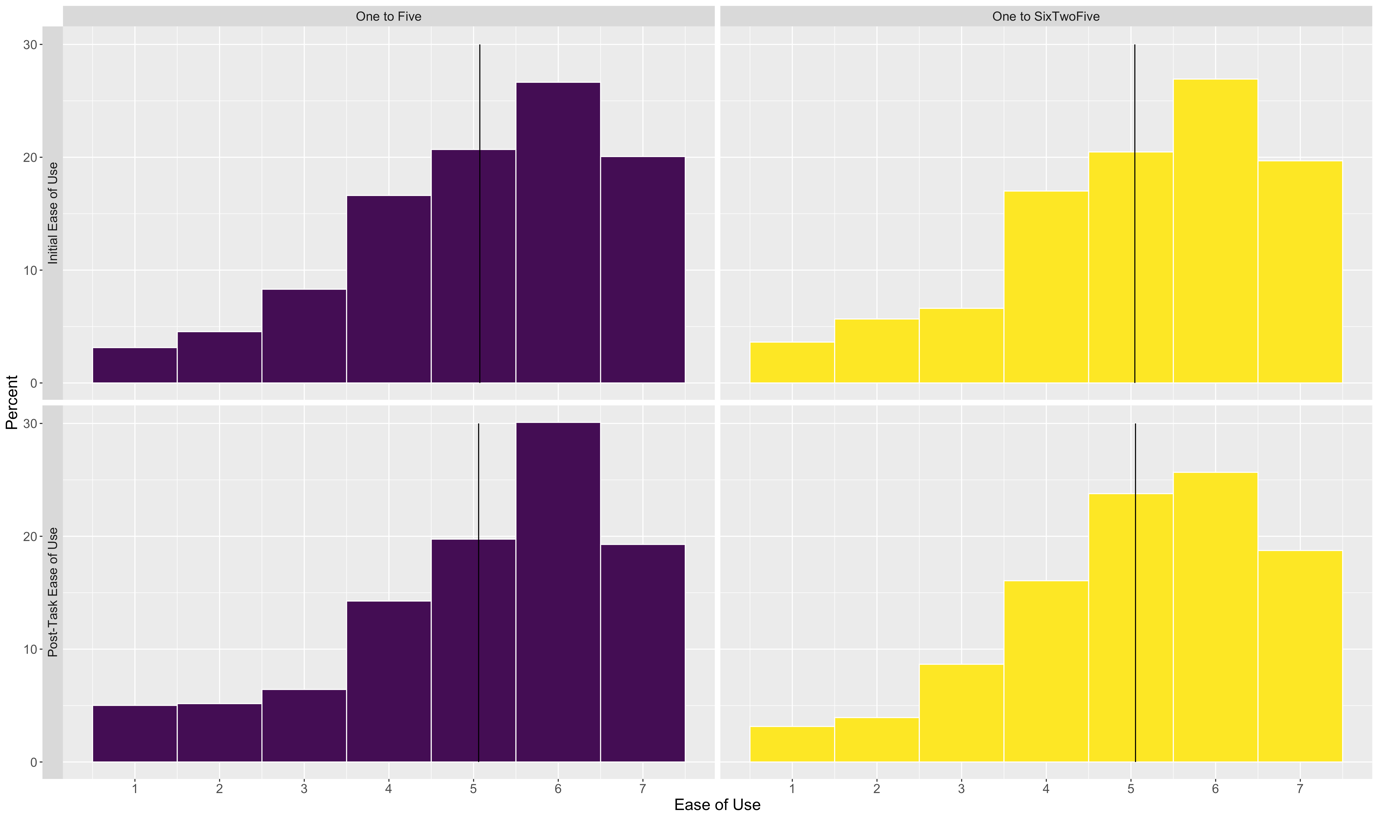


**Supplementary Fig. G13.** Experiment 2; Distribution of Pre-Task and Post-Task Ease of Use Ratings, by Key (means indicated by line)


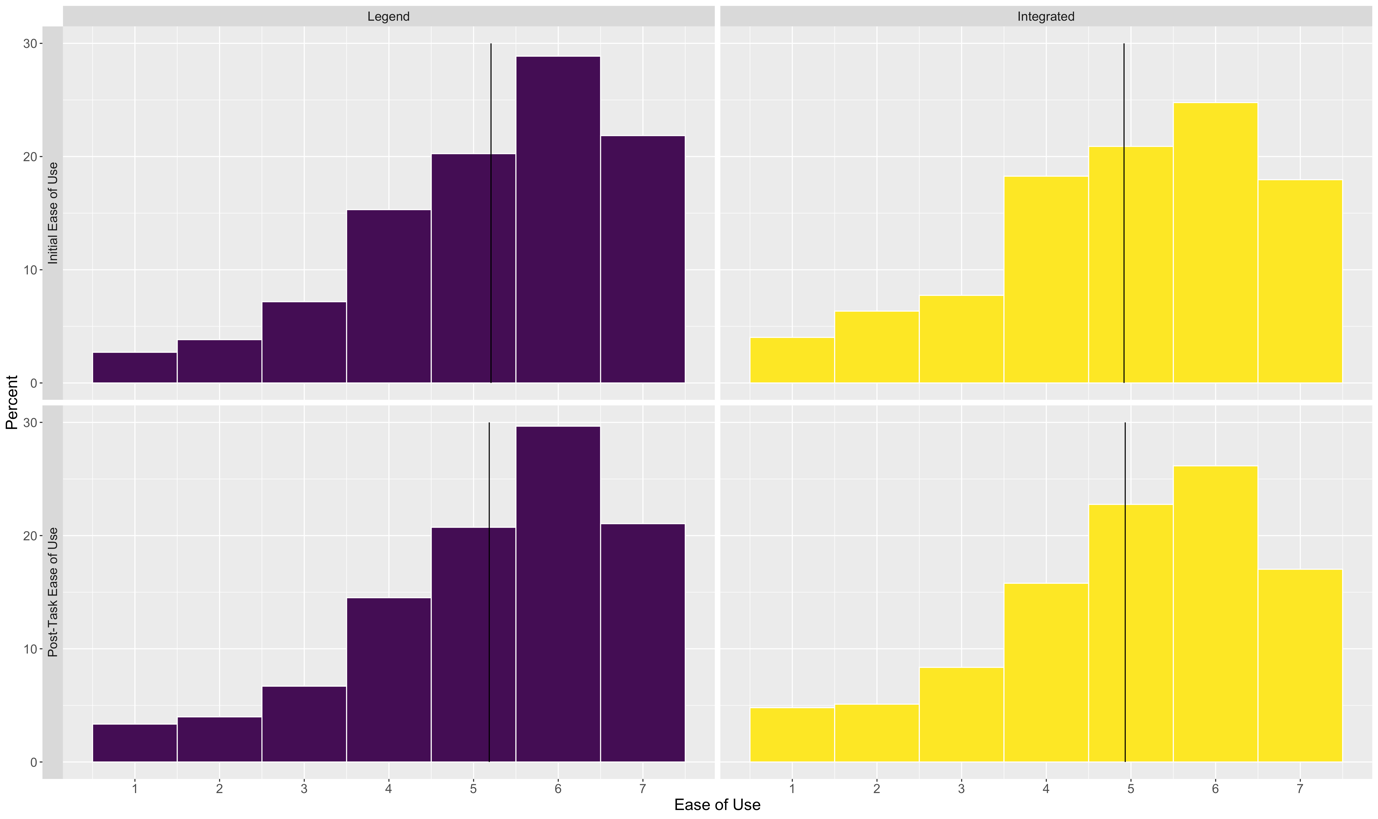


**Supplementary Fig. G14:** Experiment 2; Distribution of Post-Task Ease of ‘Confidence Using Format Oneself’ and ‘Confidence Explaining Format to Others’ Ratings, by Format (means indicated by line)


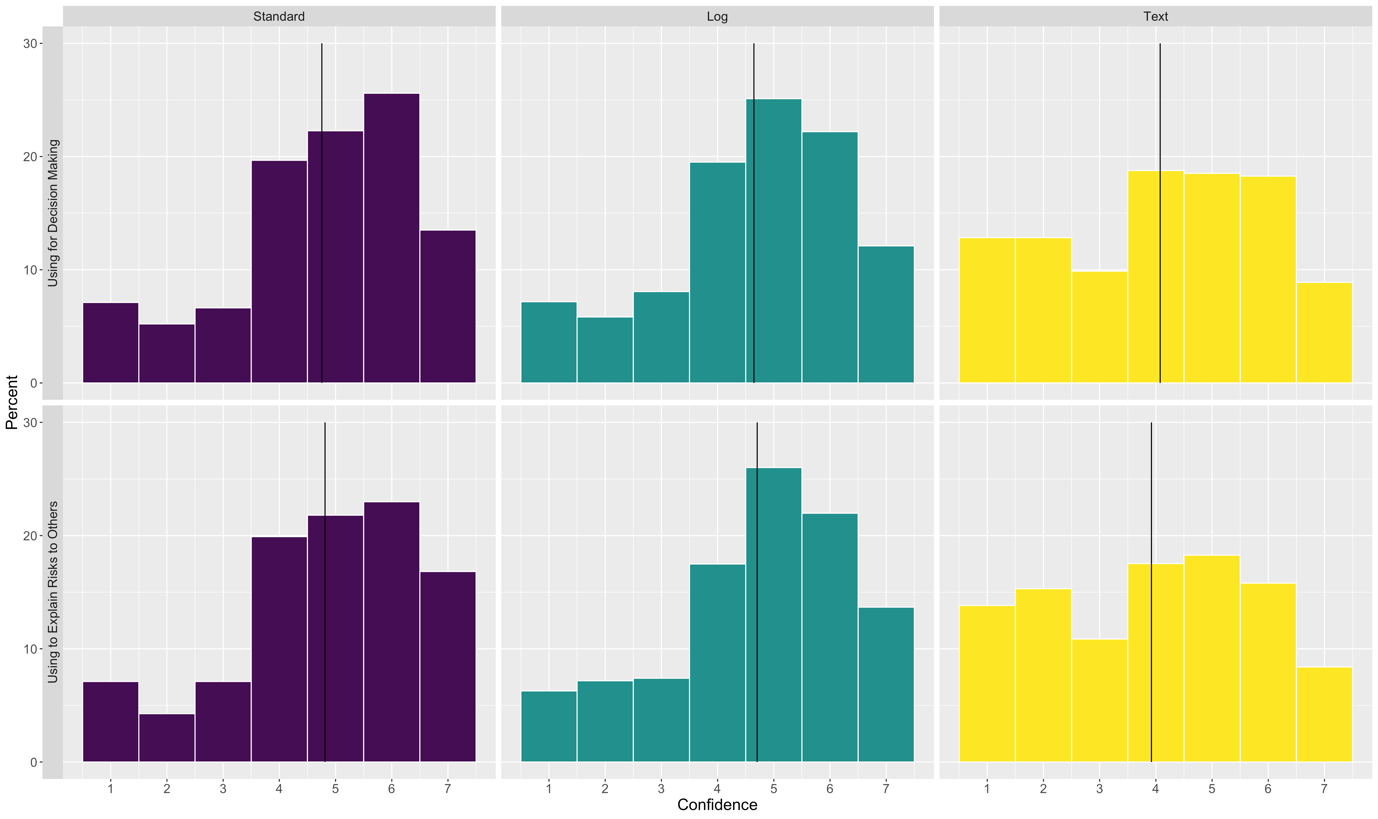


**Supplementary Fig. G15:** Experiment 2; Distribution of Post-Task Ease of ‘Confidence Using Format Oneself’ and ‘Confidence Explaining Format to Others’ Ratings, by Scale (means indicated by line)


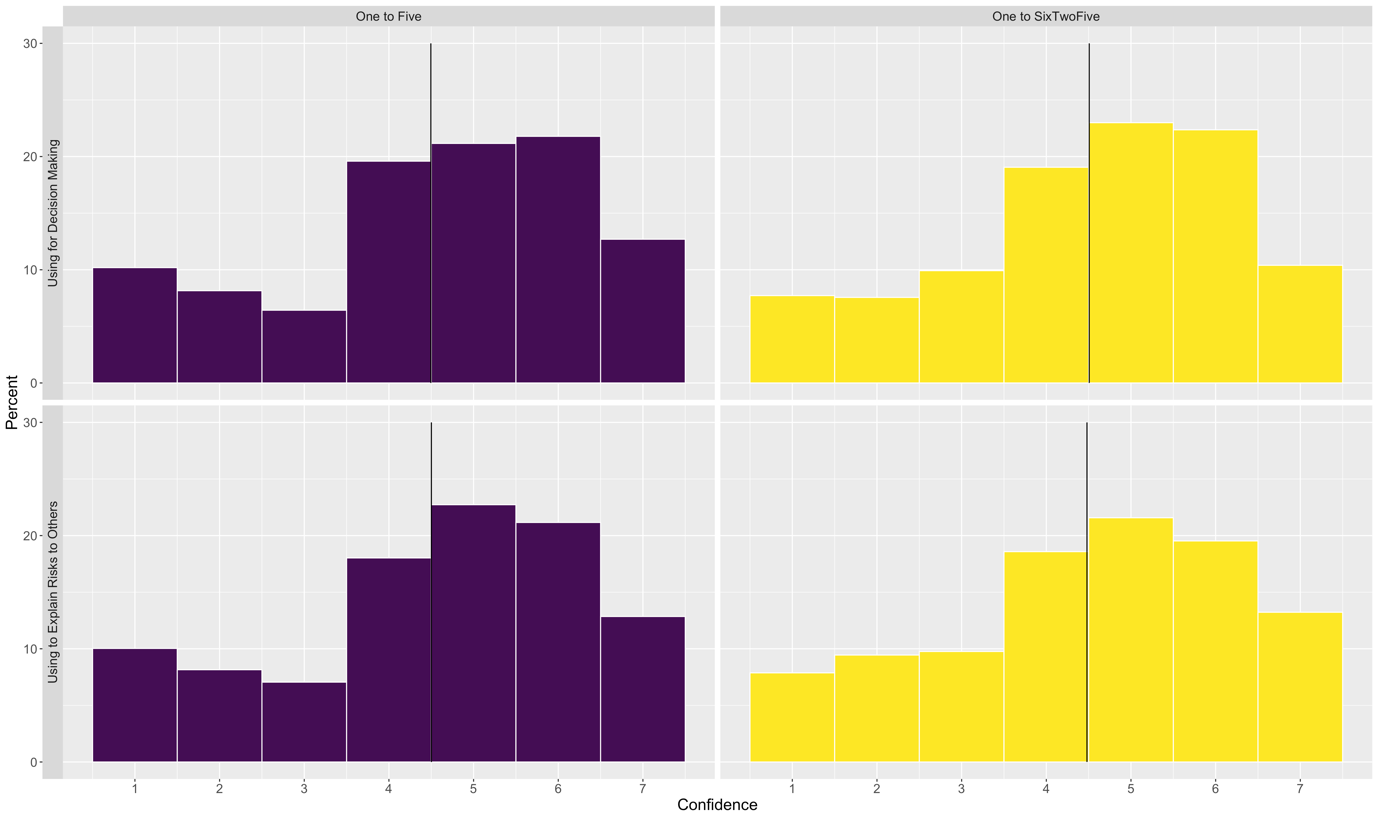


**Supplementary Fig. G16:** Experiment 2; Distribution of Post-Task Ease of ‘Confidence Using Format Oneself’ and ‘Confidence Explaining Format to Others’ Ratings, by Key (means indicated by line)


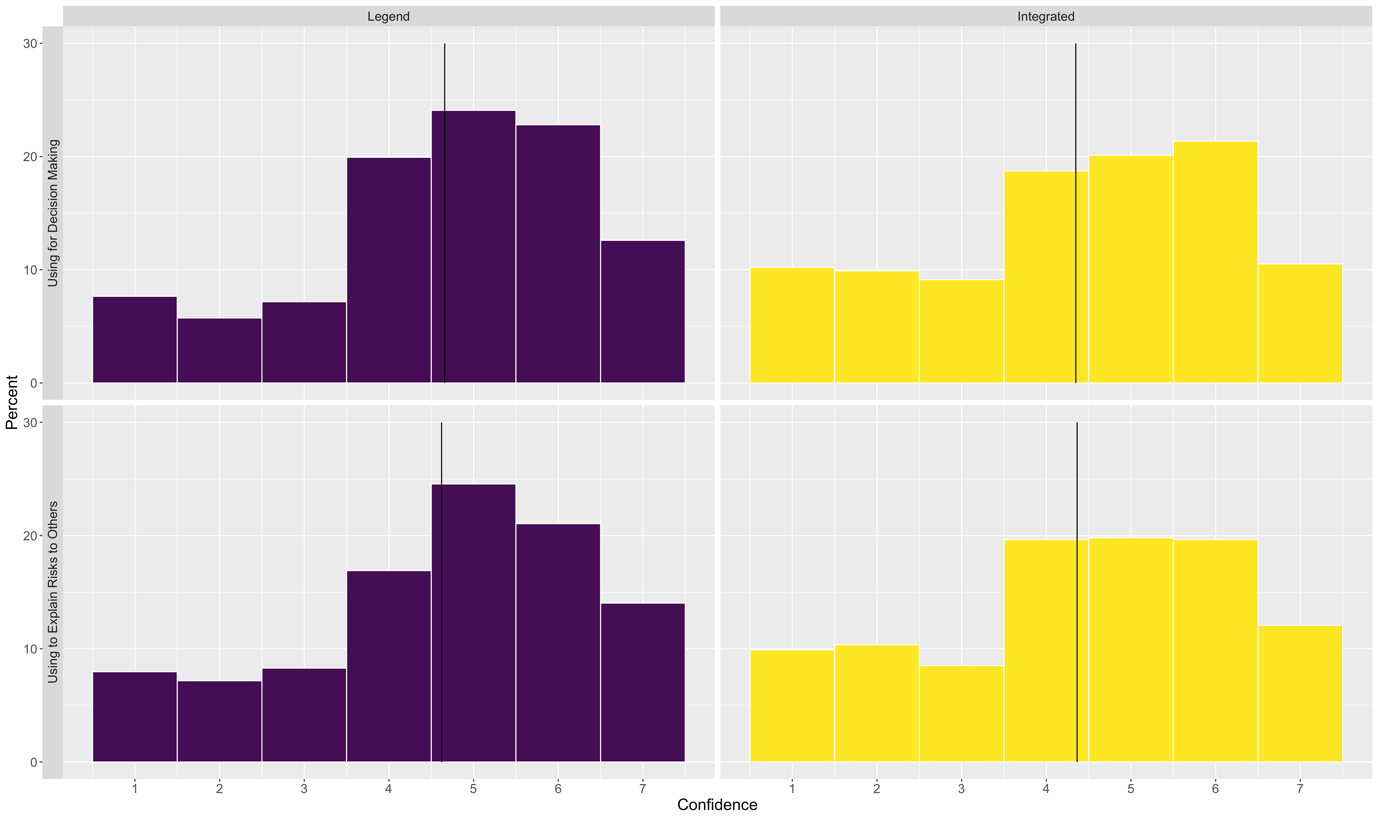

Supplement: Supplementary file 1 — Supplementary material [file RISA-42-1023-s001.docx]
